# Novel CDKL5 targets identified in human iPSC-derived neurons

**DOI:** 10.1007/s00018-024-05389-8

**Published:** 2024-08-13

**Authors:** Sean Massey, Ching-Seng Ang, Nadia M. Davidson, Anita Quigley, Ben Rollo, Alexander R. Harris, Robert M. I. Kapsa, John Christodoulou, Nicole J. Van Bergen

**Affiliations:** 1 Brain and Mitochondrial Research Group, Murdoch Children’s Research Institute, Royal Children’s Hospital, Melbourne, VIC 3052 Australia; 2 The Bio21 Institute of Molecular Science and Biotechnology Institute, University of Melbourne, Parkville, VIC Australia; 3 Murdoch Children’s Research Institute, Royal Children’s Hospital, Melbourne, 3052 Australia; 4 Walter and Eliza Hall Institute of Medical Research, Parkville, VIC 3052 Australia; 5 Electrical and Biomedical Engineering, School of Engineering, RMIT University, Melbourne, VIC Australia; 6 Aikenhead Centre for Medical Discovery, St Vincent’s Hospital Melbourne, Fitzroy, Melbourne, VIC 3065 Australia; 7 Centre for Clinical Neurosciences and Neurological Research, St. Vincent’s Hospital Melbourne, Fitzroy, Melbourne, VIC 3065 Australia; 8 Department of Medicine, St Vincent’s Hospital Melbourne, The University of Melbourne, Fitzroy, Melbourne, VIC 3065 Australia; 9 Department of Neuroscience, Central Clinical School, Monash University, Melbourne, Australia; 10 Department of Biomedical Engineering, University of Melbourne, Melbourne, 3010 Australia; 11 Victorian Clinical Genetics Services, Royal Children’s Hospital, Melbourne, VIC 3052 Australia; 12 Discipline of Child and Adolescent Health, Sydney Medical School, University of Sydney, Sydney, NSW Australia; 13 Department of Paediatrics, University of Melbourne, c/o MCRI, 50 Flemington Road, Parkville, VIC 3052 Australia

**Keywords:** CDKL5 deficiency disorder, Phosphorylation, Kinase, Neurodevelopmental disorder, Phosphoproteomics, GTF2I, PPP1R35

## Abstract

CDKL5 Deficiency Disorder (CDD) is a debilitating epileptic encephalopathy disorder affecting young children with no effective treatments. CDD is caused by pathogenic variants in Cyclin-Dependent Kinase-Like 5 (CDKL5), a protein kinase that regulates key phosphorylation events in neurons. For therapeutic intervention, it is essential to understand molecular pathways and phosphorylation targets of CDKL5. Using an unbiased phosphoproteomic approach we identified novel targets of CDKL5, including GTF2I, PPP1R35, GATAD2A and ZNF219 in human iPSC-derived neuronal cells. The phosphoserine residue in the target proteins lies in the CDKL5 consensus motif. We validated direct phosphorylation of GTF2I and PPP1R35 by CDKL5 using complementary approaches. GTF2I controls axon guidance, cell cycle and neurodevelopment by regulating expression of neuronal genes. PPP1R35 is critical for centriole elongation and cilia morphology, processes that are impaired in CDD. PPP1R35 interacts with CEP131, a known CDKL5 phospho-target. GATAD2A and ZNF219 belong to the Nucleosome Remodelling Deacetylase (NuRD) complex, which regulates neuronal activity-dependent genes and synaptic connectivity. In-depth knowledge of molecular pathways regulated by CDKL5 will allow a better understanding of druggable disease pathways to fast-track therapeutic development.

**Supplementary Information:**

The online version contains supplementary material available at 10.1007/s00018-024-05389-8.

## Introduction

The underlying disease pathogenesis of many neurological disorders is a consequence of impairments of kinase function [1]. Understanding how disrupted phosphorylation affects signalling networks that cause abnormal neuronal development and function remains a major challenge in neurobiology. CDKL5 Deficiency Disorder (CDD) is a brain disorder of young children which affects around 1:40,000 live births [2]. CDD is an X-linked disorder caused primarily by de novo pathogenic variants in the *Cyclin-Dependent Kinase-Like 5* (*CDKL5*) gene [3, 4]*.* Individuals with CDD suffer infantile-onset, drug-resistant seizures, severe neurodevelopmental impairment and profound lifelong disability [5]. CDD shares many clinical features with Rett Syndrome (RTT; caused by mutations in *MeCP2*) but is recognised as a distinct clinical disorder [6]. Both conditions carry an equivalent health burden of being quadriplegic [7], resulting in four times the annual hospital costs over that of individuals from the general population.

Cyclin-Dependent Kinase-Like 5 (CDKL5) is a protein kinase which modulates key phosphorylation events [8, 9], and is critical for the proper development and maturation of the brain [10, 11]. CDKL5 expression is most abundant in the forebrain; cerebral cortex, hippocampus, striatum and olfactory bulb [12, 13]. At the cellular level, CDKL5 is present in both glutamatergic and GABAergic neurons, with no expression detected in glial cells [14, 15], and its expression increases during neuronal maturation [16, 17]. CDKL5 is located in neuronal growth cones and regulates actin dynamics, synaptic vesicle endocytosis, excitatory synaptic stability, neurite outgrowth and dendritic spine development [16, 18, 19]. Conversely, a lack of CDKL5 expression disrupts dendritic branching and neuronal circuit connectivity [12, 16, 20, 21]. CDKL5 phosphorylates serine or threonine residues in the motif of amino acids RPX(S_p_/T_p_)[A/G/P] on the target protein [8, 9]. CDKL5 translocates between the nucleus and cytoplasm to phosphorylate specific target proteins in each compartment, thereby regulating their activity. The large C-terminal region of CDKL5 is vital for nuclear-cytoplasmic translocation [22, 23] and is postulated to impart kinase specificity.

With the recent identification of the consensus recognition site for CDKL5, unbiased phosphoproteomics studies have provided greater power in revealing important targets of CDKL5 [8, 9] than prior single-protein targeted investigations [16, 19, 24–26]. However, studies to identify CDKL5 targets have used either a non-neuronal U2O2 bone osteosarcoma cell line [9] or *Cdkl5* knockout mouse brain lysates (a mixed population of glia, astrocytes and neurons [8]). These studies revealed that CDKL5 regulates microtubule dynamics and assembly, polarity, cell signalling and cilial regulation by controlling phosphorylation of several microtubule proteins that are critical for correct neuronal architecture. In the nuclear compartment, CDKL5 phosphorylates transcriptional regulators, DNA damage repair proteins and pre-mRNA splicing factors (reviewed by [27]). Despite these advances in CDKL5 target identification, analysis of CDKL5 targets in affected human brain neurons has not yet been attempted. CDD patient-derived neurons differentiated from induced pluripotent stem cells (iPSCs) provide a valuable alternative approach to identification of new CDKL5 targets since the samples are derived from the affected cell type, human brain neurons. Here, we discovered new targets of CDKL5 identified using an unbiased phosphoproteomics study in an iPSC-derived human brain neuronal model from a CDD patient. We used our existing CDD model which utilised a patient-derived male iPSC line [*CDKL5;* c.175C > T, p.(Arg59*)] and CRISPR-Cas9 gene-corrected isogenic controls (genetically matched) [28]. We generated *bona fide* human iPSC-derived cortical neurons by dual-SMAD inhibition [29–32], which we previously demonstrated recapitulate many features of CDD, including impaired neurite outgrowth and reduced phosphorylation of EB2 [28], which is a direct phosphorylation target of CDKL5 [8, 9, 16]. We reveal perturbed phosphorylation pathways in patient-derived neurons that are critical for neuronal function. Such knowledge may identify CDKL5 brain-specific signalling and regulatory pathways underpinning CDKL5 function. It is important to note that the discovery of CDKL5 targets in human neuronal cells is a pre-requisite to identify new targeted treatments for CDD.

## Materials and methods

### Patients and cell lines

iPSC from a male patient with the *CDKL5* pathogenic variant c.175C > T [p.(Arg59*)] [6] and isogenic controls were generated and previously characterised [28]. iPSC were differentiated into neurons for 35 days of maturation as previously described [28] for RNAseq and phosphoproteomics.

### RNAseq and quantitative PCR analysis

RNA was isolated using a commercially available kit (Qiagen RNeasy® kit), quality-control checked (Qubit and TapeStation) and sequenced at the Victorian Clinical Genetics Services (Illumina TruSeq stranded mRNA library) on the NextSeq 500 with paired-end 75 bp reads and approximately 30 million fragments per sample. Fastqc was run on each fastq file, reads trimmed then aligned to the human reference genome (hg38) using STAR version 2.5.2[33] in two pass mode. Duplicate reads were removed using Picard Tools MarkDuplicates version 2.0.1 [34]. Aligned reads were summarised to genecode version 20 [35] gene-level counts using featureCounts v1.5.0 [36]. The genotype of samples was confirmed in IGV [37]. One CDKL5 p.(Arg59*) neuron sample failed genotyping QC and was excluded. Count data was analysed in R (Version 3.4.0) using edgeR glmLRT [38] method (version 3.18.1) to assess gene expression changes. Genes with a false discovery rate (FDR) of < 0.05 and a fold-change (FC) of > 2 were considered significant (Supplemental Table 1).

cDNA was synthesized (GoScript™ Reverse Transcription random hexamers master mix, Promega) and quantitative reverse-transcription PCR reactions were conducted with custom primers (Supplemental Table 2) and AccuPower® 2X Greenstar™ qPCR Master Mix (Bioneer Pacific) on a Roche LightCycler 480 II.

### Phosphopeptide enrichment of neuronal lysates

Proteins were rapidly extracted from neurons in chilled RIPA buffer [25 mM Tris pH 8.0, 150 mM NaCl, 1% NP-40, 1% sodium deoxycholate, 0.5% sodium dodecyl sulfate (SDS), 1 mM ethylenediaminetetraacetic acid (EDTA)] containing protease and phosphatase inhibitors (protease and phosphatase inhibitor mini tablets, EDTA-free, Pierce), sonicated, protein concentration estimated (BCA kit, Pierce) then stored at −80 °C. 1 mg of proteins were precipitated overnight at − 20 °C, using 5 × volume ice cold acetone. The following day the samples were centrifuged at 15,282×*g* for 10 min, pellet rinsed with ice cold acetone and centrifuged again. The pellet was solubilized in 8 M urea in 50 mM tetraethylammonium bromide (TEAB) and incubated for 30 min at 37 °C. 10 mM tris(2-carboxyethyl)phosphine (TCEP) and 55 mM iodoacetamide was added and incubated at 37 °C for 45 min. Samples were then diluted to 1 M urea with 25 mM TEAB and digested overnight in the dark at 37 °C with sequencing grade modified trypsin (Pierce, #90057) at 1:50 protein:enzyme (20 µg of trypsin per 1 mg sample). Samples were acidified to 1% (v/v) trifluoroacetic acid (TFA). Oasis HLB 60 mg cartridges were washed with 80% acetonitrile (ACN) containing 0.1% TFA, followed two washes with 0.1% TFA. Proteins were loaded onto cartridges and washed with 0.1% TFA twice then eluted with 80% ACN containing 0.1% TFA. A final BCA assay was performed for equal sample loading and 20 µg of sample was freeze-dried for total proteomics. These samples were then resuspended in a buffer containing 2% ACN, 0.05% TFA before LC–MS/MS analysis. The remaining ~ 980 µg of sample was freeze-dried and stored at −80 °C until phosphopeptide enrichment.

For phosphopeptide enrichment, TiO beads (Titansphere Phos-TiO beads, GL Sciences inc; ratio of 6:1 TiO:peptides) were washed with 50% ACN, 5% TFA and then incubated for 10 min with 2 M lactic acid in 5% TFA (washing buffer), 50% ACN (loading buffer). TiO beads in loading buffer were added to the freeze-dried phosphoproteomic peptide pellet samples and incubated for 1 h with shaking at high speed. The sample was briefly centrifuged, then loaded onto C8 zip tips (200 µl volume) and eluant passed over tip again to maximise binding.

The phosphopeptides were washed twice with loading buffer then three times with washing buffer. The phosphopeptides were eluted off with three elutions of 30 µl of elution buffer (1% (v/v) NH_4_OH, pH 11), followed by a 10 µl elution with 30% ACN. Elutions were pooled and acidified with 1 µl of formic acid per 10 µl eluent then freeze-dried and stored at −80 °C. The phosphopeptides were randomized and resuspended in 2% ACN, 0.05% TFA just before LC–MS/MS analysis.

### Mass spectrometry (MS) analyses of neuronal lysates

Samples were analysed by nanoESI-LC–MS/MS using an Orbitrap Exploris 480 mass spectrometer (Thermo Scientific) and a nanoflow reversed-phase-HPLC (Ultimate 3000 RSLC, Dionex). The LC system was equipped with an Acclaim Pepmap nano-trap column (Dinoex-C18, 100 Å, 75 µm × 2 cm) and Acclaim Pepmap RSLC analytical column (Dinoex-C18, 100 Å, 75 µm × 50 cm). The tryptic peptides were injected (1 mg) to the enrichment column at an isocratic flow of 5 µL/min of 2% v/v ACN containing 0.1% v/v formic acid for 5 min applied before the enrichment column was switched in-line with the analytical column. The eluents were 5% DMSO in 0.1% v/v formic acid (solvent A) and 5% DMSO in 100% v/v ACN and 0.1% v/v formic acid (solvent B). The flow gradient was (1) 0–6 min at 3% B, (2) 6–95 min, 3–22% B, (3) 95–105 min 22–40% B, (4) 105–110 min, 40–80% B, (5) 110–115 min, 80–80% B, (6) 115–117 min, 80–3% and equilibrated at 3% B for 10 min before the next sample injection. All spectra were acquired in positive ionization mode with full scan MS acquired from m/z 300–1600 in FT mode at mass resolving power of 120 000, after accumulating to AGC target value of 3.0E6, with maximum accumulation time of 25 ms. The RunStart EASY-IC lock internal lockmass was used. Data-dependent HCD MS/MS of charge states > 1 was performed using a 3 s scan method, at a normalized AGC target of 100%, automatic injection, a normalized collision energy of 30%, and spectra acquired at resolving power of 15 000. Dynamic exclusion was used for 20 s.

Raw files were processed with MaxQuant (version 1.6.17.0) [39] against *Homo sapiens* proteome (SwissProt, downloaded April 2019) using default search parameters (Trypsin as cleavage enzyme, cysteine carbamidomethyl as fixed modification and methionine oxidation, serine, threonine and tyrosine phosphorylation as variable modifications) and match between run option selected, with a maximum false discovery rate (FDR) of < 0.01.

### Phosphoproteomics analysis

Phosphoproteomic data was analysed with Perseus (version 1.6.1.14 and 1.6.5.0). Contaminants and reverse peptides were removed and peptides with a phosphate localization probability higher than 0.75 were kept. Phosphopeptide intensities were log_2_ transformed, annotated and phosphopeptides with valid values (in at least three samples in at least one group) were kept for statistical analysis. Intensities were normalized by subtracting the median and imputed missing values from normal distribution (0.3 width, 1.8 down shift). Significant hits were defined using Perseus Software; Two-sided Student’s *t*-test, truncated by permutation-based FDR threshold of 0.05 (250 randomizations) and S0 factor of 0.1. Gene Ontology SLIM biological process [40, 41] was used to classify protein function. STRING [42, 43] analysis with high confidence (0.900) examine protein networks. Phosphomotif enrichment was analysed with the motif enrichment programs MeMe Suite [44] and MoMo [45]. PhosphositePlus [46] and the Kinase Library [47] were used for kinase–substrate pair prediction [48]. Phosphomatics (https://phosphomatics.com/) was used for kinase-substrate enrichment (KSEA; with a *p*-value threshold of 0.05, Networkin score threshold of 5, and a M-threshold of 5) and to generate kinase-substrate network interactions.

### DNA constructs

Plasmids from Genscript (GATAD2A clone OHu17401, ZNF219 clone OHu06145, and PPP1R35 clone OHu31026) were cloned into the pcDNA5D FRT/TO plasmid with a C-terminal 3FLAG tag. GTF2I was expressed from the pEBG plasmid with N-terminal GST tag from Addgene (plasmid #22156) because endogenous GTF2I co-precipitated with GTF2I-FLAG during FLAG pulldown assay trials. Site-directed mutagenesis was used to mutate serine into alanine (QuikChange Lightning Site-Directed Mutagenesis Kit, Agilent). Full-length untagged CDKL5 (wild-type and CDKL5 kinase‐dead K42R) were obtained from MRC Protein Phosphorylation and Ubiquitination Unit (clones 55082 and 53500). Primer sequences are available in Supplemental Table 2.

### Cell transfections, whole-cell extract preparation and Western blotting

HEK293T cells were cultured in DMEM (Thermo Fisher) + 10% FCS and 1 × penicillin–streptomycin and were seeded at 1.2 × 10^6^ cells per 100 mm plate then transfected 24 h later using lipofectamine 2000 (Thermo Fisher) with a total of 3 µg of plasmid (1.5 µg + 1.5 µg for plasmid co-transfections) for 24 h. On ice, cells were washed twice in PBS then lysed in lysis buffer (50 mM Tris–HCl (pH 7.4) buffer containing 0.27 M sucrose, 150 mM NaCl, 1% (v/v) Triton X-100, 0.5% (v/v) Nonidet NP-40 and 0.1% (v/v) 2-mercaptoethanol) supplemented with a protease and phosphatase inhibitor cocktail (protease/phosphatase inhibitor cocktail #5872, Cell Signaling Technologies or Pierce protease and phosphatase inhibitor mini tablets) and benzonase (Novagen, 50U/ml) with scraping then cleared by centrifugation at 12,000*g* for 20 min. 20 µg of protein was mixed with SDS–PAGE sample buffer containing 5% (v/v) 2-mercaptoethanol before boiling at 95 °C. Samples were resolved by 4–12% Tris–glycine SDS–PAGE gradient gels (Biorad) and transferred to Amersham Hybond 0.45 μm PVDF membrane for 90 min at 100 V. The membrane was blocked in 5% (w/v) non-fat dry milk in TBS–Tween-20 (0.2% v/v) for either 1 h at RT or 4 °C overnight with diluted primary antibodies. The membrane was washed three times in TBS–Tween-20 (0.1%(v/v)), incubated with secondary antibodies in blocking buffer for 1 h, then washed three times before adding Biorad Clarity Chemiluminescent Substrate (Biorad) and capturing ECL signal on Biorad ChemiDoc MP Imaging System imager or Amersham ECL Hyperfilm. See Supplemental Table 3 for antibody information.

### Conjugation of antibody to immunoprecipitation (IP) beads

For IP, antibodies were cross-linked to IP beads and scaled according to needs. 20 µl of a 50% bead slurry (10µL settled beads, Protein G Agarose beads, Cell Signaling Technologies) were washed twice with PBS followed by centrifugation at 3341×*g* for 1 min. Dilution buffer (1 mg/mL BSA in PBS) was added at 1:1 ratio buffer:beads, mixed by rotation for 10 min, centrifuged, then wash buffer removed. Antibody (1 µg per 10 µl settled beads, either anti-FLAG or anti-GST) in dilution buffer was added to beads and incubated for 1 h at 4 °C. Beads were washed in dilution buffer followed by PBS wash at 1:1 ratio. Dimethyl pimelimidate (DMP, pH 8–9, 13 mg/ml) was freshly prepared then added to beads at 1:1 ratio and incubated with rotation at RT for 30 min then removed. Beads were washed with 10 volumes of 0.2 M triethanolamine (TEA) in PBS. The DMP and TEA steps were repeated twice. The reaction was then quenched by the addition of 50 mM ethanolamine in PBS at 1:1 ratio for 5 min, and repeated. The beads were washed with 10 volumes of PBS. Excess unlinked antibody was removed with 1 M glycine, pH 3 at 1:1 ratio and repeated. The beads were washed in cell lysis buffer three times prior to IP.

### IP for western blot and MS

For IP, HEK293T whole-cell extract was prepared and 400 µg of cell lysate was added to 20 µl of settled conjugated beads in Eppendorf LoBind tubes and incubated for 2 h at 4 °C with rotation. Beads were washed three times with 10 × volume of lysis buffer, followed by three washes of 10 ×, 20 × then 40 × volume of ice-cold PBS. After each wash beads were centrifuged at 3341×*g* for 1 min. The sample was split equally for western blot and MS.

IP samples for western blot were eluted off the 10 µl of beads by the addition of 70 µl of 3 × SDS-PAGE loading buffer (187.5 mM Tris–HCl pH 6.8, 15% SDS, 30% glycerol, 0.03% bromophenol blue and 5% 2-mercaptoethanol) and incubation at 95 °C for 5 min. 20 µl of eluant was loaded onto each gel and separated by SDS-PAGE as described above. Membranes were probed for either FLAG or GST tag, the target protein or for phosphorylated target using the Phospho-MAPK/CDK substrates antibody.

IP samples for MS were eluted off 10 µl of beads by sequential addition of 25 μL of 100 mM TEAB, 25 μL neat TFE, 0.5 μL of 10% formic acid then 2.5 μL of 200 mM TCEP then heated at 60 °C for 1 h and centrifuged at 6000 rpm. The supernatant was moved to a new tube and 10 µl of fresh 200 mM iodoacetamide (IAM, Sigma, A3221, dissolved in 25 mM TEAB) was added, vortexed then incubated at RT for 45 min in the dark. Subsequently 2.5 µl of 200 mM TCEP was added and incubated for 30 min in the dark. 300 µl of water diluted the sample then 100 µl of 100 mM TEAB added, and pH adjusted to 7.5–8.0 by the incremental addition of 0.5 M NaOH and pH measured with litmus paper. Sequencing grade modified trypsin (Pierce, #90057) was diluted to 0.1 µg/µl in 25 mM TEAB and 5 µl was added and incubated at 37 °C overnight. Then 2 µl of neat formic acid was added, centrifuged at 13,000*g* for 10 min, supernatant transferred to a new LoBind tube, freeze-dried overnight then stored at −80 °C until MS analysis. Just prior to loading, the freeze-dried pellet was resuspended in 20 µl of 2% ACN/0.05% TFA, vortexed, incubated in a sonicating water bath for 3 min, centrifuged at 13,000 g for 10 min then repeated twice. 10 µl was then loaded on for MS analysis.

For the IP experiment the MS was operated in a targeted data-dependent mode with a targeted inclusion list containing predicted phosphopeptides from GTF2I, PPP1R35, GATAD2A and ZNF219. The inclusion list consisted of mass/charge (m/z) and charge (z) of tryptic peptides predicted from an *in-silico* digest of target proteins using Skyline software [49] and peptides previously observed in public data depositories through PeptideAtlas [50] and the present study. The nanoLC separation was identical to the above method and an Orbitrap Eclipse mass spectrometer was used. Full MS1 spectra were acquired in positive mode, 120,000 resolution, AGC target of 4e5 and maximum IT time of 50 ms. A loop count of 10 on the most intense targeted peptide were isolated for MS/MS. The isolation window was set at 1.2 m/z and precursors fragmented using normalized collision energy of 30. Resolution was at 15,000 resolution, AGC target at 2e5 and maximum IT time of 110 ms. Dynamic exclusion was set to 30 s. The system performed a dependent scan on the most intense ion if no targets were found.

### Synthesis of custom phospho-antibodies

Rabbit polyclonal phosphospecific antibodies were raised against phosphorylated (*S*_p_) peptides by Covalab by triple immunisation of New Zealand White rabbits with two phosphopeptides and one peptide control. For GTF2I Ser^674^ two phosphopeptides CLQSPKRPR[S_p_]PGSNS and CKRPR[S_p_]PGS and one peptide control CLQSPKRPRSPGSNS were used. For PPP1R35 Ser^52^ two phosphopeptides CDLSLSPRPD[S_p_]PQPRH and CSPRPD[S_p_]PQP and one peptide control DLSLSPRPDSPQPRH were used. Serum samples were tested pre-immunisation and at day 53 during production for reactivity against full-length protein expressed in HEK293T cells. Final bleeds at day 72 were affinity purified by Covalab. The serum was loaded onto a column with control peptide coupled to agarose beads, retaining unmodified peptide‐specific antibodies. The flow‐through was loaded onto a column with one of the two modified peptides coupled to agarose beads, retaining modified peptide‐specific antibodies. After elution, the eluate was assayed at Covalab using ELISA against both phosphopeptides and peptide control to determine antibody immunoreactivity and specificity for the phospho-modification.

### IP for western blot with custom phospho-antibody

For IP, 1 µg of epitope antibody (FLAG or GST) was pre-incubated with 10 µl of settled beads in 1 mg/ml BSA in PBS at 4 °C overnight. Beads were washed twice with PBS and 200 µg of cell lysate was incubated for 2 h at 4 °C with rotation. The beads were washed three times with 10 × volume of lysis buffer, then three washes of 10 ×, 20 × then 40 × volume of ice-cold PBS and centrifuged at 3341×*g* for 1 min. IP samples for western blot were eluted with 70 µl of 3 × SDS-PAGE loading buffer and incubation at 95 °C for 5 min. 20 µl of eluant was loaded onto each gel, samples separated by SDS-PAGE and transferred to PVDF. Samples were probed for either FLAG or GST tag, the protein being immunoprecipitated or phosphorylated target using custom phospho**-**antibodies. See Supplemental Table 3 for antibody information.

### Statistical analysis

Statistical analyses were carried out using either a two-tailed Student’s t-test or one-way ANOVA corrected for multiple comparisons (GraphPad Prism Software, Version 8, San Diego, CA, USA). Error bars represent the standard error of the mean (± SEM) and a *p*-value of < 0.05 was considered statistically significant.

## Results

### Phosphoproteomic screening in CDKL5 human neurons reveals global perturbances to phosphorylation

**Fig. 1:**
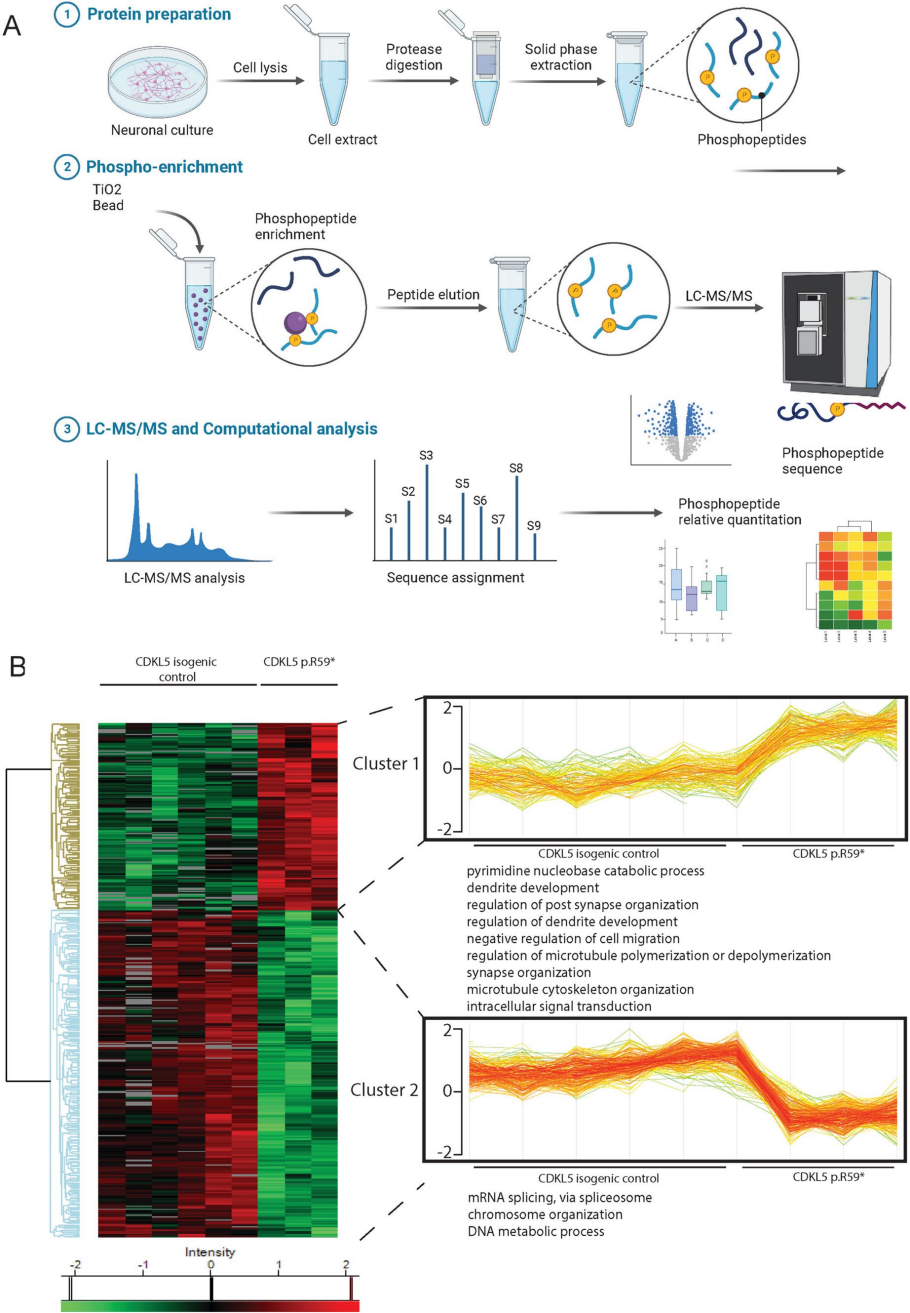
Phosphoproteomic workflow and analysis in CDKL5 human neurons. **A** Workflow for phosphoproteomic analysis in neurons. Neurons from CDKL5 p.(Arg59*) and CDKL5 isogenic controls were cultured in parallel for phosphoproteomic analysis. Neurons were lysed and protein extracts were subject to trypsin digest, peptides were extracted and phosphopeptides were enriched from each sample using TiO_2_ chromatography. Peptides were separated on a nano-HPLC and analysed by quantitative MS on an Orbitrap Mass Spectrometer. Data was analysed with MaxQuant software and visualised in Perseus software. Source data for the Fig. is available in Supplemental Materials and online. **B** Hierarchical heatmap clustering (*z*-score normalized) of significantly changed proteins from two-sided students *T*-test (BH FDR < 0.05, S0 = 0.1; *n* = 6 CDKL5 isogenic control neurons, *n* = 3 CDKL5 p.(Arg59*) neurons) between the CDKL5 p.(Arg59*) and CDKL5 isogenic control showing phosphosites identified by phosphoproteomic analysis. Horizontal tree indicates 9 independent samples. Vertical tree indicates the 454 significant phosphosites identified. Phosphorylation proteins were separated into two clusters, and each cluster was interrogated by Gene Ontology (GO) analysis. Two of the proteins clusters showing shared GO clusters were bracketed and individual samples were plotted as profile plots of log2-transformed normalized intensities for phosphoproteins in CDKL5 p.(Arg59*) and CDKL5 isogenic controls

To identify the phosphoproteomic changes and search for new CDKL5 phosphorylation targets, we used unbiased quantitative LC–MS/MS established for mouse cortical neurons [51, 52] which enabled the identification of new CDKL5 targets in human neurons (Fig. 1A, Supplemental Figure 1, Supplemental Table 4). A total of 4271 unique phosphosites were identified, and 454 phosphosites considered significant (Fig. 1B), of which 290 phospho-sites were enriched in isogenic controls. Most phosphorylation events occurred on serine residues, consistent with reports that CDKL5 deficiency predominantly affects serine phosphorylation [9].

**Fig. 2:**
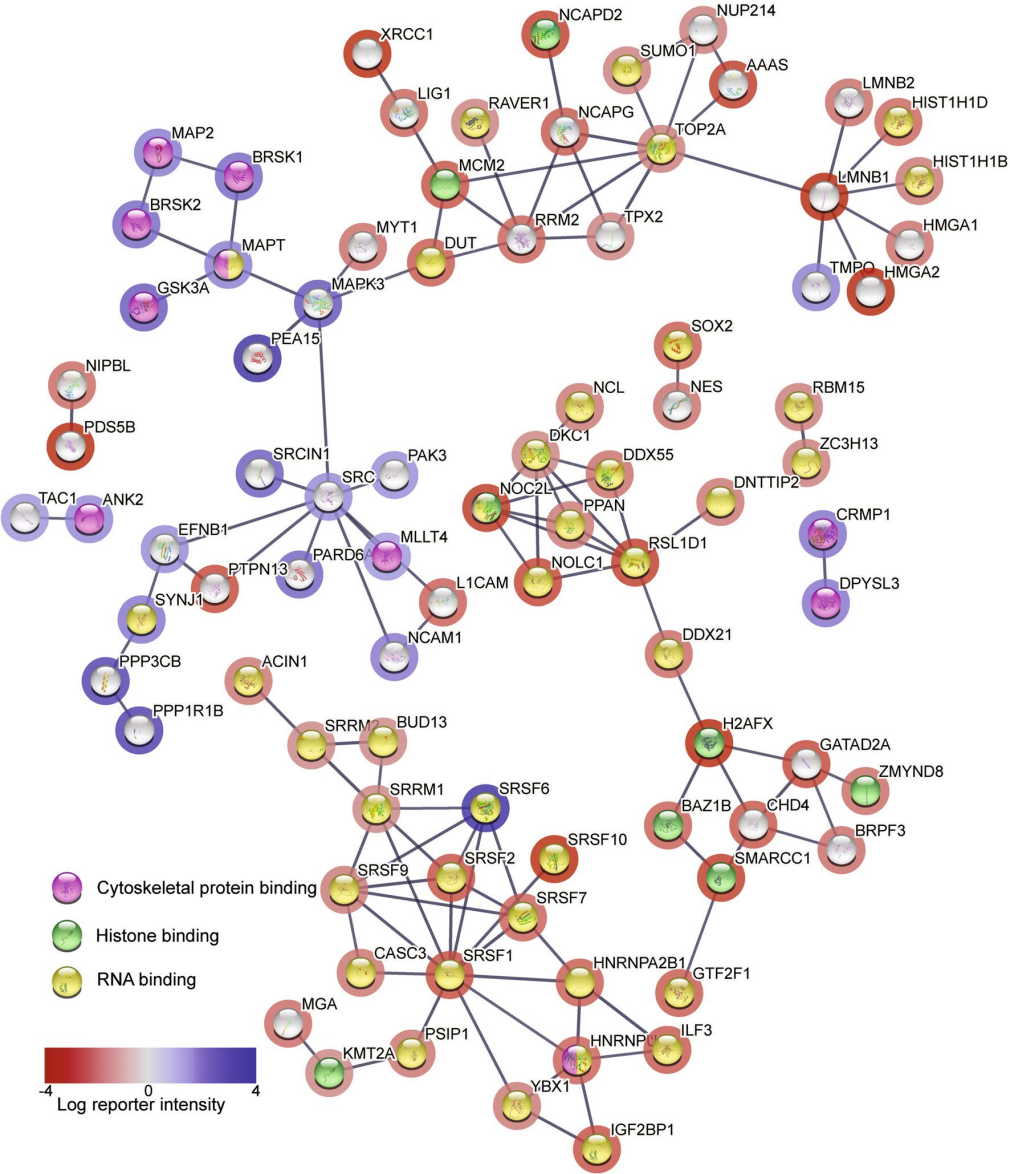
Global phosphoproteomic STRING network analysis in CDKL5 human neurons. STRING analysis (http://www.string-db.org) derived protein–protein interaction networks for phosphoproteins that are differentially regulated in CDKL5 p.(Arg59*) compared to CDKL5 isogenic control neurons. The network nodes represent proteins. Log reporter intensity is from CDKL5 p.(Arg59*)/CDKL5 isogenic control data presented in volcano plot in Fig. 4A, so blue represents higher in CDKL5 p.(Arg59*) and red represents higher in CDKL5 isogenic control. Reporter intensity is indicated by a blue or red circle around the protein name. Interaction score in STRING is set at the highest confidence (0.900), and major gene ontology groups highlighted in the centre of the circle, with cytoskeletal proteins (pink centre), histone-binding proteins (green centre) and RNA binding proteins (yellow centre) indicated

Heatmap and hierarchical clustering data (Fig. 1B, left side), separated significantly changed phosphosites into two clusters (Fig. 1B, right side) and Gene Ontology (GO) biological process analysis revealed protein function in each cluster. Cluster 1, with higher phosphorylation in CDKL5 p.(Arg59*) neurons (Fig. 1B) was enriched for pyrimidine nucleobase catabolic processes, dendrite and synapse organization, cell migration, regulation of microtubule dynamics and organization, and intracellular signal transduction. Cluster 2, representing higher phosphorylation in CDKL5 isogenic control neurons (Fig. 1B), was enriched for mRNA splicing, chromosome organization and DNA metabolic processes. STRING protein cluster grouped phosphorylation events into three major classes; of cytoskeletal protein binding, histone binding and RNA binding (Fig. 2). Cytoskeletal proteins including MAPT, MAP1A, MAP1B, MAP2, NCAM1 and DCX were more highly phosphorylated in CDKL5 p.(Arg59*) neurons. Histone and RNA binding proteins had increased phosphorylation in CDKL5 isogenic controls, including six Serine/Arginine Rich Splicing Factor (SRSF) proteins.

**Fig. 3:**
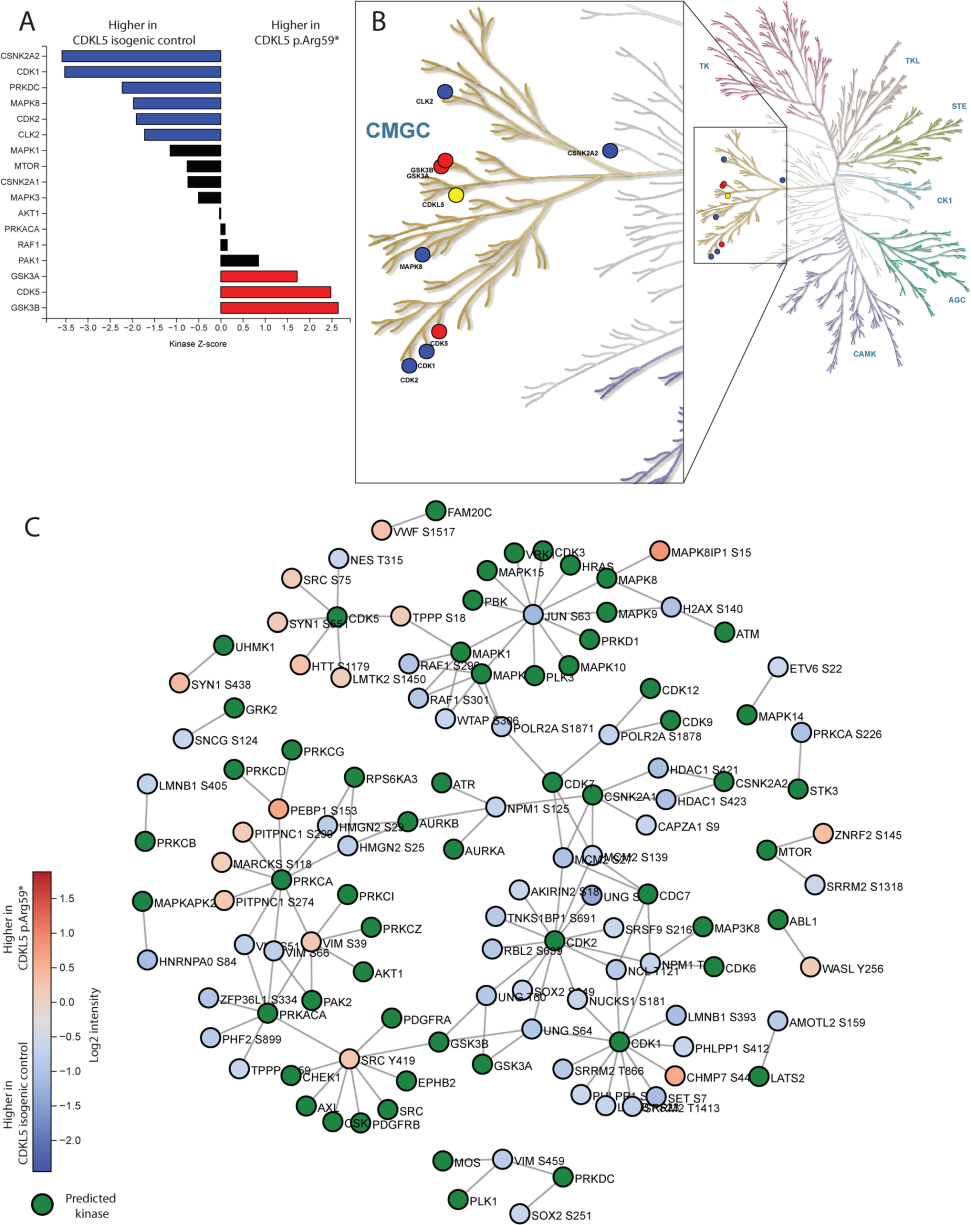
Kinome profiling and kinase-substrate interaction in CDKL5 neurons. Phosphoproteomic data was analysed in Phosphomatic.com to determine disrupted kinase activity in CDKL5 neurons. **A** KSEA analysis provides information on signalling cascades that are altered in CDD subsequent to pathogenic variants in CDKL5. KSEA analysis identifies kinases that are associated with phosphorylation sites in a data set that substantially differ in abundance between two treatment groups. The KSEA *Z*-score assesses the statistical significance of inferred activities, by normalizing the total log-fold change of substrates with the standard deviation of the log-fold changes of all sites in the dataset. Blue bars represent enrichment of the phospho-sites in CDKL5 isogenic control neurons (*Z*-scores less than 0) and red bars represent enrichment of the phospho-sites in CDKL5 p.(Arg59*) neurons (Z-score greater than 0). **B** Kinases from KSEA were mapped onto the human kinase map and circles represent enrichment of kinases that phosphorylate phospho-sites from KSEA analysis. Blue circles are higher in CDKL5 isogenic control neurons and red circles are higher in CDKL5 p.(Arg59*) neurons. CDKL5 is indicated in yellow. **C** The phosphoproteomic network displays specific relationships between the substrates in CDKL5 isogenic controls compared to CDKL5 p.(Arg59*) neurons and known upstream kinases based on data from PhosphoSitePlus and Signor. Network diagrams represent substrates of the active data group as coloured circles (blue is higher in CDKL5 isogenic control and red is higher in CDKL5 p.(Arg59*) neurons) and known upstream kinases as green circles. Colour-coding of substrates represents mean fold-change between the two groups

Kinase-Substrate Enrichment Analysis (KSEA) predicted the upstream kinases disrupted in CDKL5 p.(Arg59*) neurons (Fig. 3A). There was enrichment of GSK3α, GSK3β and CDK5 signalling in CDKL5 p.(Arg59*) neurons, whilst in CDKL5 isogenic control neurons there was enrichment of CSNK2A2, CDK1, CDK2, PRKDC, MAPK8 and CLK2 signalling. All of these kinases, including CDKL5 are within the CMGC kinase family (Fig. 3B) and particularly, GSK3A and GSK3B are closely related to CDKL5 in the human kinome phylogenetic tree. Of the 454 significantly altered phosphosites, only 48 sites (~ 10%) had a reported kinase in PhosphoSitePlus (Supplemental Table 5). The kinase-substrate (K–S) relationships were represented in a network diagram (Fig. 3C) which demonstrated disruptions to kinase signalling cascades in CDKL5 p.(Arg59*) neurons.

### Enrichment of phosphorylation motif identifies potential new neuronal targets of CDKL5

**Fig. 4:**
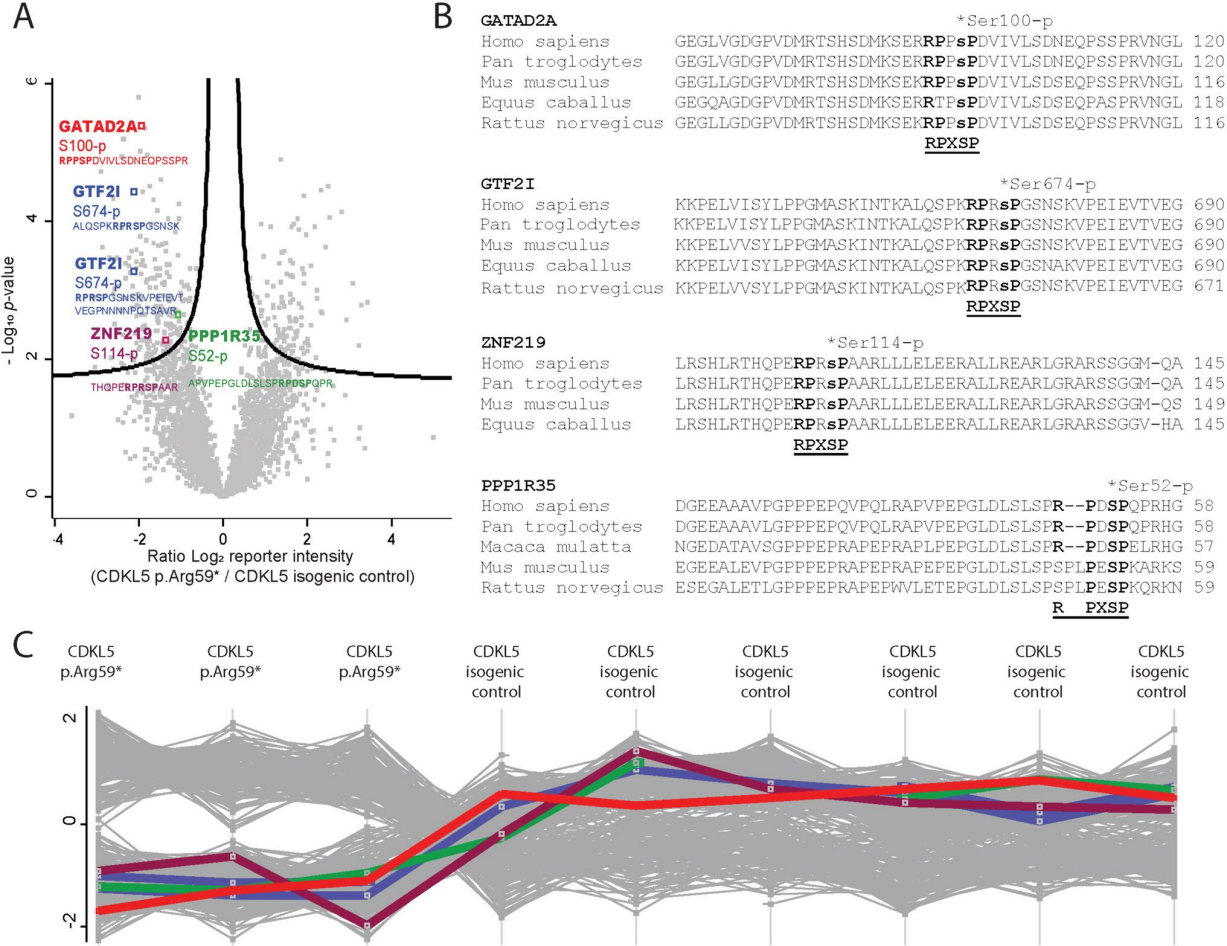
Identification of potential specific CDKL5 phosphotargets in CDKL5 human neurons. **A** Label free quantitative phospho-MS (LFQPMS) of phosphorylated CDKL5 neuronal proteins (left = higher in wild-type), highlighting sites containing the CDKL5 consensus motif. Volcano plot shows significantly changed proteins from Perseus analysis (BH FDR < 0.05, S0 = 0.1; *n* = 6 CDKL5 isogenic control neurons, *n* = 3 CDKL5 p.(Arg59*) neurons). **B** Conservation of phosphorylated sites in proteins containing the CDKL5 motif. **C** Phosphorylation level of targets in each sample were consistent between replicate samples. Each coloured line represents phosphorylation levels of the four targets identified in volcano plot in Fig. 4A (PPP1R35 in green, GTF2I in blue, GATAD2A in red and ZNF219 in purple)

The most highly enriched phosphomotif in the phosphoproteomic dataset was PxS_p_P (where ‘X’ is any residue), with a 27-fold enrichment (*p*-value of 4.60 × 10^–6^, Supplemental Table 6), followed by S_p_P with a 26-fold enrichment (*p*-value of 6.40 × 10^–6^). The combined enriched motif was RPXS_p_PXE which corresponds to the CDKL5 consensus phosphomotif RPX(S_p_/T_p_)[A/G/P] [8, 9]. In our dataset there were 4 protein targets that contained the complete CDKL5 consensus phosphomotif (Fig. 4) with phosphosignal elevated in CDKL5 isogenic control neurons. The four proteins and phosphorylated residue were GATAD2A Ser^100^, General Transcription factor II-I (GTF2I, also known as TFII-I) Ser^674^ (Uniprot P78347, or S^633^ in isoform 2; P78347-2), ZNF219 Ser^114^ and PPP1R35 Ser^52^ (Fig. 4B). Ser^674^ in GTF2I was identified from two different phosphopeptides (Supplemental Table 7). Importantly no phosphopeptides containing the consensus CDKL5 motif were identified elevated in CDKL5 p.(Arg59*) neurons. The phosphorylation motifs were highly conserved (Fig. 4B, Supplemental Figure 2).

We identified the predicted kinase at each phosphorylation site in our dataset (Supplemental Table 7) [47]. For GTF2I Ser^674^, CDKL5 is predicted to be the 6th most likely kinase, for PPP1R35 Ser^52^, CDKL5 is the 2nd most likely kinase, for GATAD2A Ser^100^ CDKL5 had a lower Log_2_ score and ranked as the 14th most likely kinase whilst for ZNF219 Ser^114^ CDKL5 was the 10th most likely kinase.

### PPP1R35 as a new potential CDKL5 phospho-target

**Fig. 5:**
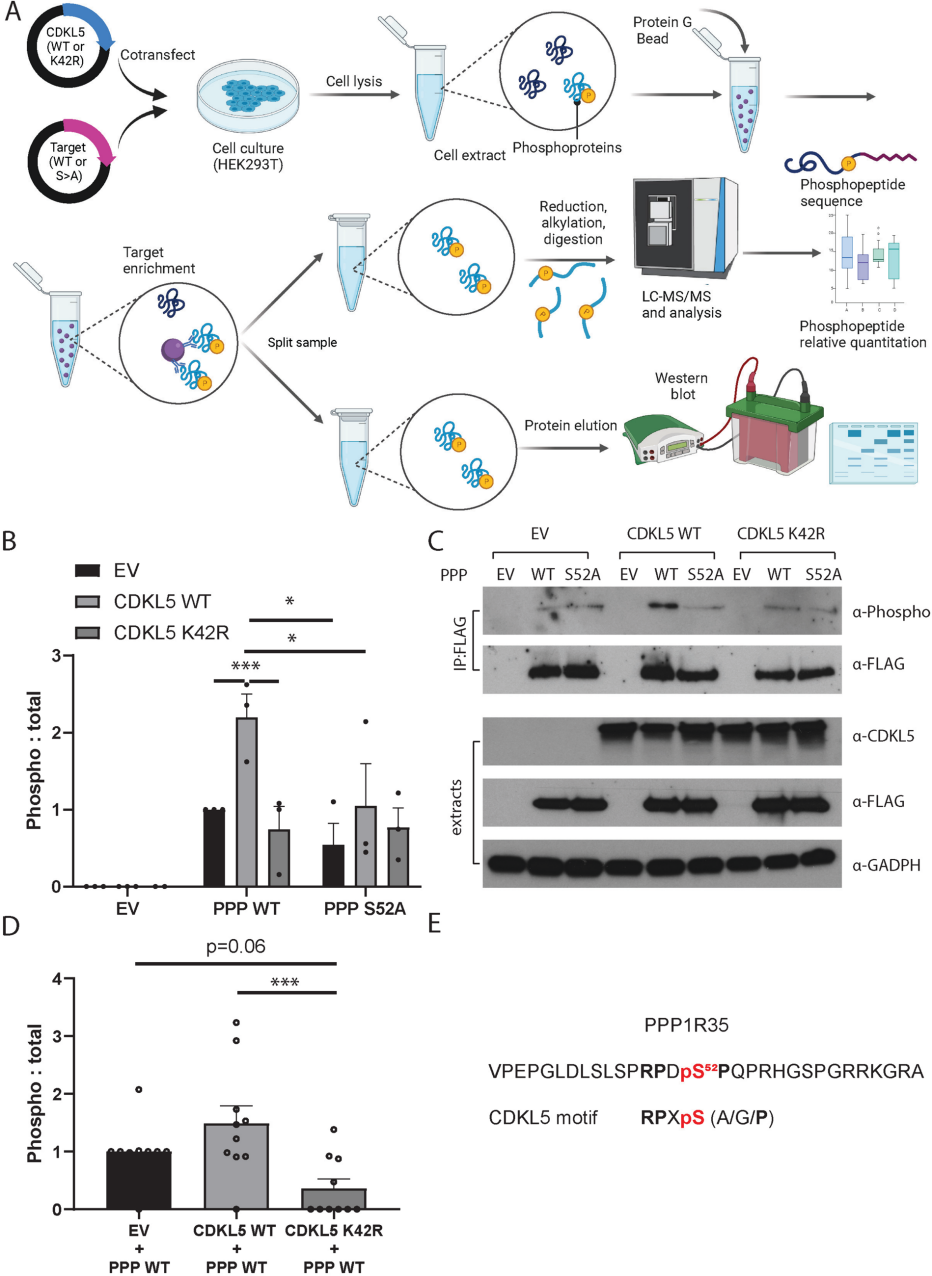
Orthogonal validation approach to demonstrate that CDKL5 phosphorylates PPP1R35 at Ser^52^ in human cells. **A** HEK293T cells were co-transfected with CDKL5 WT or CDKL5 K42R and PPP1R35 (PPP) WT or PPP1R35 S52A, where serine is converted to an alanine. Cells were then lysed and the target was immunoprecipitated with an antibody against the epitope FLAG tag. Immunoprecipitated proteins were split between quantitative phospho-MS and phospho-western blotting. **B** Anti‐FLAG immunoprecipitates were probed with Anti-MAPK/CDK -Phospho (top panel), and anti-FLAG (bottom panel). Empty vector (EV) lysates were used as a negative control. Quantification of three independent experiments showed a significant increase in the phosphorylation levels of PPP1R35 WT when co-expressed with CDKL5 WT compared to either empty vector or CDKL5 kinase dead (K42R) mutant. Site-directed mutagenesis (PPP1R35 S52A) did reduce overall phosphorylation levels when comparing PPP1R35 WT to PPP1R35 S52A. Data is representative of 3 independent samples from 3 independent experiments. Data is mean ± SEM. *P < 0.05, **P < 0.01. One-way ANOVA with Dunnett’s multiple comparisons test. **C** Lysate extract sets from co-transfection experiments were probed with anti-CDKL5 (top panel), anti-FLAG (middle panel) and anti-GAPDH (bottom panel). The expression patterns indicate that the co-transfection experiments were successful. Three independent experiments were completed, and one representative experiment is shown. **D** Phosphorylation of PPP1R35 WT was higher in HEK293T cells co-transfected with CDKL5 WT compared to co-transfected with CDKL5 K42R or empty vector. Phospho-peptides were quantified using LC–MS/MS intensity of phosphopeptides and was normalised to the label-free quantification of PPP1R35. Data is representative of 10 independent samples from six independent experiments. Data is mean ± SEM. ***P < 0.001. One-way ANOVA with Sidak’s multiple comparisons test. **E** Phosphopeptide detected from PPP1R35 that is phosphorylated by CDKL5

To confirm if PPP1R35 is a bona-fide target of CDKL5, we co-expressed either wild-type PPP1R35 (PPP-WT) or PPP1R35 mutant Ser^52^Ala (SA) protein with either CDKL5 wild-type (CDKL5-WT) or CDKL5 kinase-dead (K42R) protein, enriched protein by IP and determined phosphorylation levels by MS and western blotting, as previously described[9]. We utilised an antibody that recognises phospho-MAPK/CDK substrates with a phospho-motif (PXS*P or S*PXR/K) that would detect proteins containing the CDKL5 motif (Fig. 5A).

When CDKL5 WT was co-expressed with PPP1R35 WT, phosphorylation levels were significantly higher than when co-expressed with CDKL5 K42R (Fig. 5B, C). Site-directed mutagenesis which abolished the phosphoserine site of PPP1R35 significantly reduced phosphorylation levels.

We then generated a custom antibody against the PPP1R35 Ser^52^ site which was tested and validated for specificity to PPP1R35 Ser^52^ (Supplemental Figures 3, 4B, 5C). This custom phospho-antibody also confirmed that highest phosphorylation occurred when PPP1R35 WT was co-expressed with CDKL5 WT, and phosphorylation was significantly lower when co-expressed with CDKL5 K42R (Supplemental Figure 6).

Quantitative phospho-MS from IP samples above (Supplemental Table 8) demonstrated the highest phosphorylation of PPP1R35 at Ser^52^ occurred when CDKL5 WT was co-expressed with PPP1R35 WT and was significantly lower when co-expressed with CDKL5 K42R (Fig. 5D). Site-directed mutagenesis (S52A) completely abolished the phospho-signal on PPP1R35 (Supplemental Fig. 7A) even though protein input was similar (Supplemental Fig. 7B and Fig. 5C, α-FLAG lane). The PPP1R35 peptide identified by MS in both neuronal phosphorylation data and IP data (APVPEPGLDLSLSPRPDSPQPR) correctly identified the phosphoserine Ser^52^ within the CDKL5 motif (Fig. 5E). Together the data provides evidence that CDKL5 directly phosphorylates PPP1R35 at Ser^52^.

### GTF2I as a new potential CDKL5 phospho-target

**Fig. 6:**
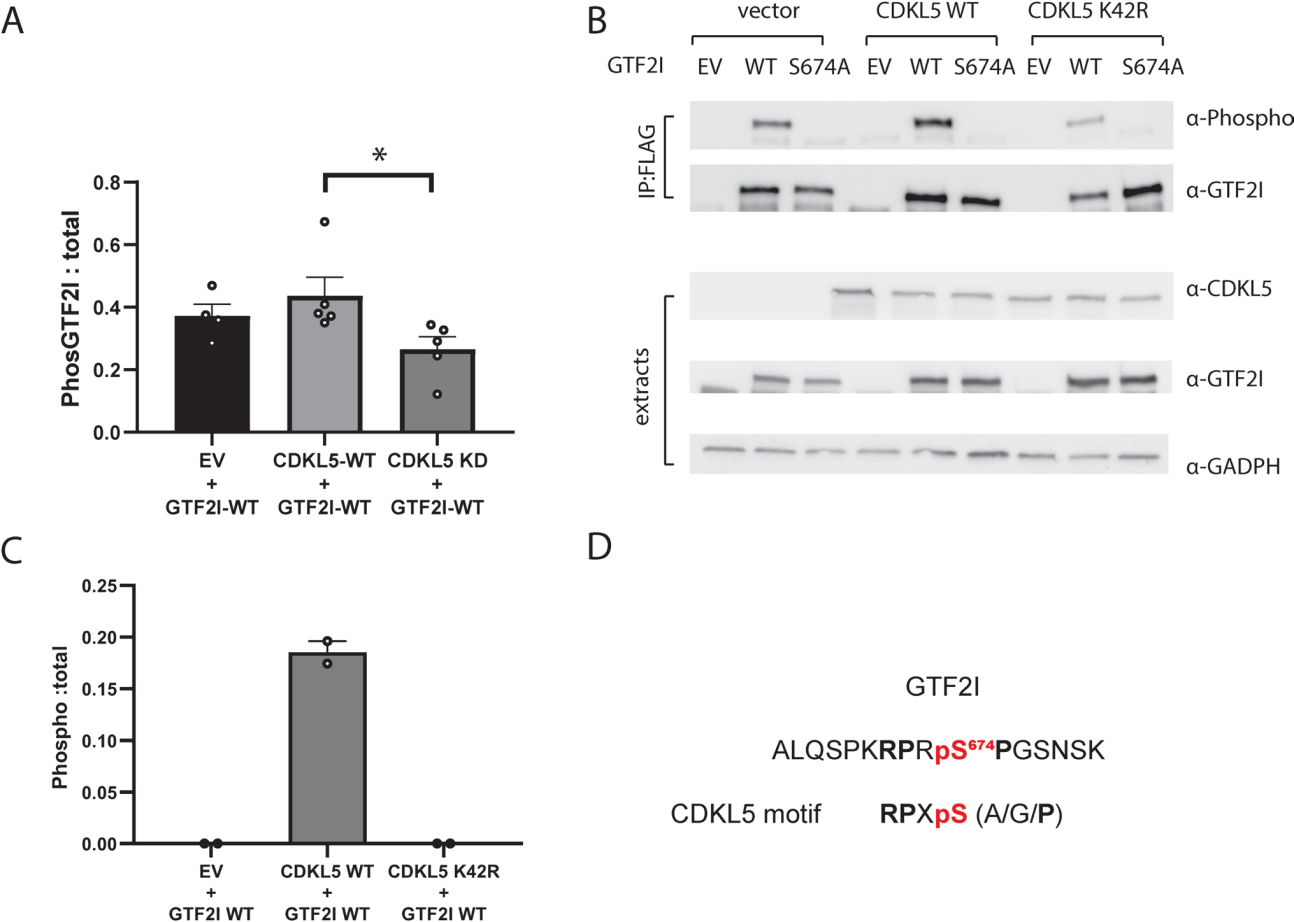
Orthogonal validation approach to demonstrate that CDKL5 phosphorylates GTF2I at Ser^674^ in human cells. **A** Quantification of three independent experiments showed a significant difference in the phosphorylation levels in GTF2I when co-expressed with CDKL5 WT compared to either empty vector (EV) or CDKL5 kinase dead (K42R) mutant. Site-directed mutagenesis of GTF2I at Ser^674^ to Ala (SA) completely abolished phosphorylation levels and was not quantifiable. Data is representative of 5 independent samples from 5 independent experiments. Data is mean ± SEM. *P < 0.05. **B** Lysate extract sets from co-transfection experiments were probed with anti-CDKL5 (top panel), anti-GTF2I (middle panel) and anti-GAPDH (bottom panel). Five independent experiments were completed and the expression patterns indicate that the co-transfection experiments were successful. Data is mean ± SEM. *P < 0.05. One-way ANOVA with Dunnett’s multiple comparisons test. **C** Phosphorylation of GTF2I WT was only detected in HEK293T cells co-transfected with CDKL5 WT compared to co-transfected with CDKL5 K42R or empty vector. Phospho-peptides were quantified using LC–MS/MS intensity of phosphopeptides was normalised to the label-free quantification of GTF2I. The phosphorylation event was detected in two of six independent experiments. **D** Phosphopeptide detected from GTF2I that is phosphorylated by CDKL5

Using the same co-IP approach as for PPP1R35, we assessed whether CDKL5 directly phosphorylates GTF2I at Ser^674.^ The antibody that detects phospho-MAPK/CDK substrates only produced a faint signal but still revealed the highest phosphorylation level when GTF2I WT was co-expressed with CDKL5 WT (Supplemental Fig. 8). We then generated and validated custom phospho-specific antibody for GTF2I Ser^674^ (Supplementary Figs. 4A, 5A, and 9). With this antibody we showed highest phosphorylation when GTF2I WT was co-expressed with CDKL5 WT, and phosphorylation levels were significantly lower when co-expressed with CDKL5 K42R (Fig. 6A, B). Quantitative MS only detected phosphorylation events in immunoprecipitated GTF2I WT when co-expressed with CDKL5 WT and phosphorylation was never detected when GTF2I WT was co-expressed with CDKL5 K42R or empty vector control (Fig. 6C, Supplemental Table 9). GTF2I protein input levels were similar for MS analysis (Supplemental Fig. 10B). This evidence supports the conclusion that CDKL5 directly phosphorylates GTF2I at Ser^674^.

The custom phospho-antibodies generated for both GTF2I and PPP1R35 were not able to detect endogenous phosphorylation of GTF2I or PPP1R35 in neuronal lysates due to high background signal despite multiple attempts (data not shown).

### Downstream targets of GTF2I regulation are altered in CDKL5 p.(Arg59*) neurons

GTF2I regulates of a suite of genes primarily in the brain and phosphorylation may modulate transcriptional activity[53, 54]. RNAseq Differential Expression (DE) analysis identified 379 genes significantly downregulated in CDKL5 p.(Arg59*) neurons, and 99 significantly upregulated when compared to CDKL5 isogenic controls (Supplemental Table 1).

We compared the list of DE genes in CDKL5 to previously reported RNAseq datasets of GTF2I-regulated genes. This included data from *Gtf2i*-knockout mouse brain, brain biopsies from Williams-Beuren Syndrome (WS) patients with a microdeletion including *GTF2I*, WS iPSC [53, 54] and WS fibroblasts [55]. We identified 20 genes dysregulated in CDKL5 p.(Arg59*) neurons that were also dysregulated in WS brain biopsies and WS iPSC [53–55]. Of these 20 genes, 10 genes were downregulated in both CDKL5 p.(Arg59*) neurons and WS (Supplemental Table 10) and one gene, *BEND4* was upregulated in both (Supplemental Fig. 11). Comparison of our DE list to *Gtf2I*-knockout mouse cortex [54] revealed an additional 3 genes, two of which were similarly downregulated both in the mouse model and in our CDKL5 p.(Arg59*) neurons. Additionally, 2 genes, the gap junction connexin family gene *Gjc2* and the claudin family gene *Cldn11*, were downregulated in the *Gtf2I* knockout mouse cortex [54]. Although the *Gjc2* and *Cldn11* genes were unchanged in our CDKL5 neurons, other genes in the gap junction connexin family, *GJB7*, *GJA1* and the claudin family, *CLDN1, CLDN4* and *CLDN6* were downregulated in CDKL5 p.(Arg59*) neurons (Supplemental Table 10). GTF2I represses *BEND4* expression [53] and both our RNAseq (Supplemental Table 10) and qPCR analysis (Supplemental Fig. 11) showed increased *BEND4* in CDKL5 p.(Arg59*) neurons. BEND4 protein was not detected in our proteomics dataset [28]. There was no change in *GTF2I* transcript or protein abundance [28] implying that any changes to GTF2I are post-translational, not due to intrinsic changes in transcript expression or protein levels.

### Dysregulation of nucleosome remodelling deacetylase (NuRD)-complex related genes

Orthogonal studies harnessing the HEK293T overexpression system to determine whether CDKL5 phosphorylates GATAD2A and ZNF219 did not support phosphorylation of these targets by CDKL5 (Supplemental Figs. 12 and 13). Phospho-MS was unable to reliably capture the phosphorylation event on immunoprecipitated ZNF219. Quantitative phospho-MS analysis of GATAD2A Ser^100^ showed unchanged levels of GATAD2A phosphorylation, whether co-expressed with empty vector, CDKL5 WT or CDKL5 K42R (Supplemental Fig. 13D). Despite this, both GATAD2A and ZNF219 associate with the NuRD complex, a neuronal gene regulatory complex [56, 57]. GATAD2A and CHD4 are part of the core, and ZNF219 is an accessory subunit. A suite of neuronal genes are dysregulated in the *Chd4*^*−/−*^ mouse brain [57]. We compared the DE genes in our CDKL5 neuronal RNAseq dataset to the *Chd4*^*−/−*^ mouse cortex and identified 11 genes that were downregulated in both datasets (Supplemental Table 11).

## Discussion

### Phosphoproteomic perturbations in CDKL5 p.(Arg59*) neurons

Our unbiased phosphoproteomics study identified changes in global kinase signalling in CDKL5 p.(Arg59) neurons. KSEA analysis infers kinase-substrate (K-S) relationships, however only ~ 5% of phosphosites have a reported upstream kinase and only ~ 20% of known kinases are well-represented in source databases [58]. Although we identified 454 significantly altered phosphosites, KSEA only recognised 48 K-S relationships (~ 10%). Despite this, all significantly disrupted kinases were in the CMGC family, indicating that compensatory signalling pathways may be occurring in the absence of CDKL5 or that CDKL5 has a critical role in multiple signalling cascades. KSEA demonstrated enrichment of CSNK2A2, CDK1, CDK2, PRKDC, MAPK8 and CLK2 signalling in CDKL5 isogenic controls. CSNK2A2 is the alpha catalytic subunit of the protein kinase enzyme, casein kinase 2 (CK2) that phosphorylates hundreds of substrates with a specific phosphorylation motif [58] and is often overrepresented in phosphoproteome datasets. CDK1 and CDK2 have a critical role in maintaining cell cycle and apoptosis, and cytoskeletal dynamics, processes which are critical to neuronal development [59].

There was enrichment of GSK3α, GSK3β and CDK5 signalling in CDKL5 p.(Arg59*) neurons. CDKL5, GSK3α and GSK3β are closely related in the human kinome, and both CDKL5 and GSK3B are from a specific branch of the CMGC kinases that regulate cilium length [60]. Overexpression of CDK5 and GSK3β cause cytoskeletal abnormalities and microtubule hyperphosphorylation, including MAPT (Tau) [61, 62]. We identified hyperphosphorylation of MAPT in CDKL5 p.(Arg59*) neurons, which has also been reported in *Cdkl5-KO* mice [63]. GSK3β is expressed in the central nervous system and negatively regulates neurological developmental processes [64]. Loss of CDKL5 enhances AKT/ GSK3β signalling [21], conversely inhibition of GSK3β signalling rescues developmental phenotypes in CDD mouse models [65, 66]. GSK3β overactivation is associated with numerous neurological/developmental conditions and is an attractive therapeutic target [67]. Furthermore, GSK3β inhibition was identified in a high-throughput drug screen for CDD [68]. Targeting GSK3β may be a logical therapeutic approach for CDD.

We identified increased phosphorylation of cytoskeletal and microtubule-related proteins (MAPs), notably MAPT (Tau), MAP1A, MAP1B, MAP2, NCAM1 and DCX, in CDKL5 p.(Arg59*) neurons. MAPs are critical to neuronal development, maturation and maintenance and post-translational phosphorylation affects microtubule stability, function and localisation. For example, phosphorylation releases DCX, MAPT, MAP2, MAP4 and MAP6 from microtubules [69], and their increased phosphorylation in CDKL5 p.(Arg59*) neurons may indicate an intrinsic instability of the neuronal microtubule network. Several MAPs are direct targets of CDKL5 including EB2 (MAPRE2) and MAP1S [8, 9] and broadly CDKL5 regulates microtubule dynamics [8, 9, 70–72].

In CDKL5 p.(Arg59*) neurons there was decreased phosphorylation of histone, RNA-binding and splicing proteins, including six SRSF proteins which facilitate pre-mRNA splicing and RNA regulation [73]. CDKL5 is localised to nuclear speckles, enriched in pre-mRNA splicing factors [74], and may indirectly or directly phosphorylate several chromatin remodeling and DNA methylation proteins including MeCP2 [10, 23, 25], HDAC4 [24], DNMT1 [75], SOX9 [76] and SMAD3 [77].

We searched for new targets with the CDKL5 consensus motif RPX(S_p_/T_p_)[A/G/P] [8, 78] and identified four new potential CDKL5 targets; PPP1R35, GTF2I, ZNF219 and GATAD2A. These targets have cellular functions consistent with the known functions of CDKL5. PPP1R35 is a putative regulator of Protein Phosphatase 1 (PP1) and is a centrosomal-associated protein that regulates centriole number and cilial length. GTF2I interacts with the basal transcriptional machinery and links specific cellular responsive activator complexes, particularly in brain tissues. GATAD2A and ZNF219 belong to the NuRD complex, which regulates neuronal activity-dependent genes [56], and synaptic connectivity [57]. Kinase-substrate evidence was strongest for CDKL5 being the kinase phosphorylating PPP1R35 and GTF2I, whilst GATAD2A and ZNF219 ranked lower.

### PPP1R35 (PP1 regulatory subunit 35) a cilial and centrosomal protein as a new target of CDKL5

Our phosphoproteomics data from human iPSC-derived neurons and subsequent validation by IP and MS supports PPP1R35 as a new CDKL5 target. PPP1R35 is required for centriole to centrosome conversion [79], cell cycle progression [80], for centriole duplication, and in ciliogenesis [80, 81]. Regulation of cell cycle and cilial function is critical for neurogenesis and neuronal maturation [82]. Human congenital ciliopathies are caused by defects in centriolar proteins and are the underlying cause of some cases of microcephaly [83, 84]. PPP1R35 is a candidate ciliopathy gene, associated with microcephaly, intellectual disability and global developmental delay [80, 85, 86]. *Ppp1r35*-/- mice had developmentally delayed embryogenesis and severe morphological defects, with a lack of primary cilia [81].

PPP1R35 directly interacts with CEP131 [87], a direct CDKL5 phosphorylation target [9]. CEP131 is a centrosomal protein responsible for formation of primary cilia [88]. Rotatin (RTTN) is another PPP1R35 interacting protein and is associated with microcephaly and centriole length regulation [87]. RTTN regulates the structure of primary cilia and centrioles, and in mature neurons may affect neuronal migration [89]. PPP1R35 is critical for centriole elongation and recruiting microtubule-binding elongation machinery, processes that are impaired in CDKL5 [8, 9]. CDKL5 is present at the centrosome, is involved in ciliogenesis [60, 71, 90] and in regulating proper cilial length [91]. This suggests that CDKL5 may regulate cilia through direct phosphorylation of both CEP131 and PPP1R35.

Intracellular CDKL5 localisation is partly driven by Dyrk1a-mediated phosphorylation [92] and, upon NMDA-receptor driven stimulation, can be dephosphorylated by PP1, which confines CDKL5 to the cytoplasm [93]. Interestingly, PPP1R35 is a regulatory subunit of PP1 [94] and most PP1-regulatory proteins inhibit PP1 phosphatase activity. Phosphorylation of PP1-regulatory proteins (e.g. PPP1R35) provides an additional level of PP1 regulation. It could be reasonably speculated that in response to external stimuli such as NMDA-receptor stimulation PPP1R35 is part of a feedback loop regulating PP1 phosphorylation, which in turn regulates CDKL5.

The Ser^52^ residue of PPP1R35 is one of three highly conserved, and closely located serine residues often identified in large-scale phosphoproteomic studies, highlighting their biological significance [95–97]. Mutagenesis of any of these three serines did not impair PPP1R35 interacting with RTTN or centriole formation [87]. However adjacent to the conserved serine residues at amino acids 77–81 is a putative PP1 binding and regulation site [94, 98] whilst amino acids 103–253 regulate centrosome biogenesis [79]. Consequently, it remains to be determined whether CDKL5-mediated phosphorylation of PPP1R35 at Ser^52^ is critical for PP1 regulation, centrosomal biogenesis or cilia length.

### GTF2I (General transcription factor II–I), a gene regulatory protein, as a new target of CDKL5

The second novel CDKL5 target we identified was GTF2I, with phosphorylation occurring at Ser^674^. According to Uniprot, Ser^674^ in the canonical protein (Uniprot P78347) corresponds to S^633^ in isoform 2 (P78347-2). Site-directed mutagenesis of Ser^633^ in isoform 2 (Ser^674^ in the canonical protein) impairs GTF2I activity on the c-*fos* promoter, but does not affect DNA-binding of GTF2I [99]. GTF2I is highly expressed in the brain, predominantly in neurons [100] and controls axon guidance, calcium signalling, neuronal apoptosis [101], neuronal differentiation [102] and cell cycle by regulating a suite of specific neuronal development genes [53, 103, 104]. The activity and specificity of GTF2I is regulated by phosphorylation at serine or tyrosine residues, and a number of kinases have been implicated in GTF2I regulation including Btk, c-Src, JAK2, ERK/MAPK and cGMP. Specifically, ERK is reported to phosphorylate GTF2I at the same site (Ser^674^) as we report for CDKL5 [99]. GTF2I is a multifactorial kinase target involved in many signalling pathways, and it is possible that there is more than one kinase that phosphorylates at this site. Phosphorylation may provide a rapid and reversible transactivation of GTF2I and expression of GTF2I-regulated genes.

GTF2I binds to core promoter elements and upstream elements to regulate transcription by coordinating the formation of a multiprotein complex at the cFOS promoter, linking specific signal responsive activator complexes [105]. The cFOS promoter is a highly responsive immediate-early gene promoter that responds transiently from a range of extracellular stimuli and is essential in determining neuronal survival [106]. When hippocampal cFOS expression is eliminated, neuronal apoptosis is increased and mice experience heightened excitability and severe kainic-acid induced seizures [107]. Interestingly, several studies report severely reduced cFOS expression in V1 cortex of *Cdkl5*^*−/y*^ mutants [108] with impairment in the whisker tactile stimulation-induced neuronal activation [109], and reduced cFOS expression persisted with age [110]. If CDKL5 regulates phosphorylation of GTF2I, resulting in altered cFOS activity, dysregulation of this pathway may explain the seizure-like phenotype and increased neuronal apoptosis in the absence of CDKL5 [77, 111].

GTF2I also directly interacts with PolII and Elongin A (ELOA) in response to stress, and regulates transcription of the *DNMT1* and *EFR3A* genes [112]. DNMT1 is phosphorylated by CDKL5 [75], although not within the CDKL5 consensus motif [27]; however ELOA is directly phosphorylated by CDKL5 in the known CDKL5 phosphorylation motif in response to DNA damage [78].

GTF2I and the functionally related GTF2IRD1 are two of up to 28 genes deleted in the microdeletion syndrome Williams-Beuren Syndrome (WS; OMIM #194,050). WS is characterised by craniofacial dysmorphology, intellectual disability and hyper-sociability and specifically GTF2I deletion explains many of the social and cognitive features of WS [113]. Mouse models of WS (either single or combined GTF2I/GTF2IRD1 gene knockouts) showed transcriptional activity changes restricted to chromatin modifier and synaptic protein genes [114], and the function of GTF2I was enriched for regulating signal transduction proteins, specifically phosphorylation and WNT signalling [114]. Despite a 50% knockdown of GTF2I in the mouse model, transcriptional changes were still quite subtle [114]. In autism spectrum disorders, individuals with GTF2I have hyposociability [115], the opposite to WS patients who are hypersocial. Mice with a single copy of Gtf2i had increased axonal outgrowth, whilst mice with 3 copies of Gtf2i had decreased axonal outgrowth [116]. This suggests a gene-dose effect of GTF2I could disrupt neuronal transcriptional circuits during early developmental stages [103]. This is in line with other animal models of chromatin modifier genes (e.g. MeCP2) with relatively modest transcriptional dysregulation [117–119], implying that these genes require precise transcriptional dosing for correct neuronal development. It is likely that chromatin modifying proteins including GTF2I and MECP2 regulate the dynamics of neuronal gene expression, and fine-tuning their activity via CDKL5-mediated phosphorylation regulates activity and specificity.

Upon decreased GTF2I phosphorylation in CDKL5-deficent neurons, we may anticipate impaired transcriptional and regulatory activity of GTF2I, as demonstrated upon site-directed mutagenesis of Ser^674^ in GTF2I [99]. A study of both WS brain biopsies and *Gtf2I* knockout mouse cortex revealed a suite of downregulated genes likely regulated by GTF2I [54]. Interestingly, we demonstrated 19 genes dysregulated in CDKL5 p.(Arg59*) neurons were also dysregulated in GTF2I models. Specifically, *BEN domain containing 4* (*BEND4*) is a gene directly repressed by GTF2I [53], and *BEND4* and *GTF2I* levels are negatively correlated in the human brain [53]. We showed a significant increase in *BEND4* expression in CDKL5 p.Arg59* neurons, without changes to GTF2I transcript or protein levels. It is possible that if CDKL5 does not actively phosphorylate GTF2I the transcriptional repressor activity of GTF2I is impaired, and dysregulation of a suite of genes, including *BEND4* expression occurs. Little is known on the function of BEND4, but it is part of a family of neural repressor transcription factors [120]. Patients with missense variants in *BEND4* had acute neurological deterioration following infections, lost most social and language skills, which was associated with a pontine cyst, calcification, and cerebellar atrophy [121]. Our findings suggest that CDKL5 may regulate BEND4 and a suite of other neuronal genes via GTF2I phosphorylation.

### Are ZNF219 and GATAD2A CDKL5 phosphotargets?

Our phosphoproteomics data from CDKL5 neurons identified GATAD2A and ZNF219 as potential CDKL5 targets. Although our orthogonal overexpression studies in HEK293 failed to reveal significant differences in phosphorylation by CDKL5, overexpression of components of the NuRD complex may disrupt endogenous cell signalling pathways, or potentially prevent correct NuRD subunit composition by interfering with the native NuRD stoichiometry [122], which can vary in composition in a cellular context [123] and tissue specific manner [124]. Interactions with core subunits including GATAD2A [125, 126] and non-canonical proteins such as ZNF219 [127] as well as post-translational modifications [124] regulate NuRD complex activity.

The ZNF219 Ser^114^ phosphorylation site facilitates interaction between ZNF219 and the NuRD complex [127] which may impact cell-specific regulation of downstream target genes. While the mouse homologue of GATAD2A, also known as p66α, was phosphorylated at Ser^96^ (corresponds to human Ser^100^) in response to neuronal activation, histone modifications and neuronal rewiring [128].

The NuRD complex is a critical epigenetic regulator during neuronal development [57, 129], and maintains experience-dependent neuronal plasticity [123]. CDKL5-mediated phosphorylation of NuRD proteins including ZNF219 and GATAD2A may ensure accurate transcriptional activity of the NuRD complex in the brain. Although our orthogonal studies were not able to determine whether CDKL5 directly phosphorylates GATAD2A or ZNF219, we assessed whether there were transcriptional responses which may support dysregulation of the NuRD complex. Interestingly, our RNAseq data in CDKL5 p.(Arg59*) neurons identified 11 genes that were also downregulated in *Chd4*^*−/−*^ mice [122]. Knockout of *Chd4,* a key subunit of the NuRD complex, dysregulates a suite of genes in the mouse cortex [57]. CDKL5 may be a key kinase that modulates phosphorylation of ZNF219 and/or GATAD2A and subsequent NuRD activity, and further investigation is clearly warranted. Researchers have obtained more reliable results utilising an in vitro rabbit reticulocyte lysate expression system when studying the NuRD complex to better recapitulate native complex stoichiometry, which could be a future approach to validate these potential CDKL5 targets [130].

## Conclusion

For the first time our data demonstrates that pathogenic variants in *CDKL5* may inhibit phosphorylation of PPP1R35, GTF2I, GATAD2A and ZNF219. We performed phosphoproteomics in tissue models of iPSC-derived neurons from CDD patients, compared to isogenic controls and validated protein phosphorylation in transfected HEK293T cells. These human-derived models are more relevant to understanding CDD disease relevant molecular pathways than animal models. However, tissue models are themselves distinct entities with different cellular structure, epigenetics and microenvironments, which may alter molecular pathways in comparison to the patients from which the iPSCs were derived. Further validation of the identified protein markers should be performed in more complex iPSC-derived brain organoids and in primary tissue from patients wherever possible. Uncovering the molecular pathways regulated by CDKL5 may enable development of new targeted treatments for CDD and associated disorders.

## Supplementary Information

Below is the link to the electronic supplementary material.

Supplementary file1 (DOCX 7770 KB)

Supplementary file2 (TXT 1459 KB)

Supplementary file3 (TXT 18697 KB)

Supplementary file4 (TXT 248 KB)

Supplementary file5 (TXT 153 KB)

## Data Availability

The de-identified raw data is available upon request.

## Abbreviations

CDKL5: Cyclin-dependent kinase-like 5
CDD: CDKL5 deficiency disorder
NuRD: Nucleosome remodelling deacetylase complex
RTT: Rett syndrome
iPSC: Induced pluripotent stem cells
WS: Williams-Beuren syndrome
KSEA: Kinase-substrate enrichment analysis
