# Correction: Novel CDKL5 targets identified in human iPSC-derived neurons

**DOI:** 10.1007/s00018-024-05421-x

**Published:** 2024-09-10

**Authors:** Sean Massey, Ching-Seng Ang, Nadia M. Davidson, Anita Quigley, Ben Rollo, Alexander R. Harris, Robert M. I. Kapsa, John Christodoulou, Nicole J. Van Bergen

**Affiliations:** 1 Brain and Mitochondrial Research Group, Murdoch Children’s Research Institute, Royal Children’s Hospital, Melbourne, VIC 3052 Australia; 2 The Bio21 Institute of Molecular Science and Biotechnology Institute, University of Melbourne, Parkville, VIC Australia; 3 Murdoch Children’s Research Institute, Royal Children’s Hospital, Melbourne, 3052 Australia; 4 Walter and Eliza Hall Institute of Medical Research, Parkville, VIC 3052 Australia; 5 Electrical and Biomedical Engineering, School of Engineering, RMIT University, Melbourne, VIC Australia; 6 Aikenhead Centre for Medical Discovery, St Vincent’s Hospital Melbourne, Fitzroy, Melbourne, VIC 3065 Australia; 7 Centre for Clinical Neurosciences and Neurological Research, St. Vincent’s Hospital Melbourne, Fitzroy, Melbourne, VIC 3065 Australia; 8 Department of Medicine, St Vincent’s Hospital Melbourne, The University of Melbourne, Fitzroy, Melbourne, VIC 3065 Australia; 9 Department of Neuroscience, Central Clinical School, Monash University, Melbourne, Australia; 10 Department of Biomedical Engineering, University of Melbourne, Melbourne, 3010 Australia; 11 Victorian Clinical Genetics Services, Royal Children’s Hospital, Melbourne, VIC 3052 Australia; 12 Discipline of Child and Adolescent Health, Sydney Medical School, University of Sydney, Sydney, NSW Australia; 13 Department of Paediatrics, University of Melbourne, c/o MCRI, 50 Flemington Road, Parkville, VIC 3052 Australia

**Correction: Cellular and Molecular Life Sciences (2024) 81:347**

10.1007/s00018-024-05389-8

In the original version of this article, the given and family names of Van Bergen NJ were incorrectly structured.

The name was displayed correctly in all versions at the time of publication.

The original article has been corrected.

