# Supplementary material for: Novel CDKL5 targets identified in human iPSC-derived neurons
Source: Cell Mol Life Sci. 2024 Aug 13;81(1):347. doi: 10.1007/s00018-024-05389-8 (PMC11335273; doi:10.1007/s00018-024-05389-8)
Supplement: Supplementary file 1 — Supplementary file1 (DOCX 7770 KB) [file 18_2024_5389_MOESM1_ESM.docx]

**
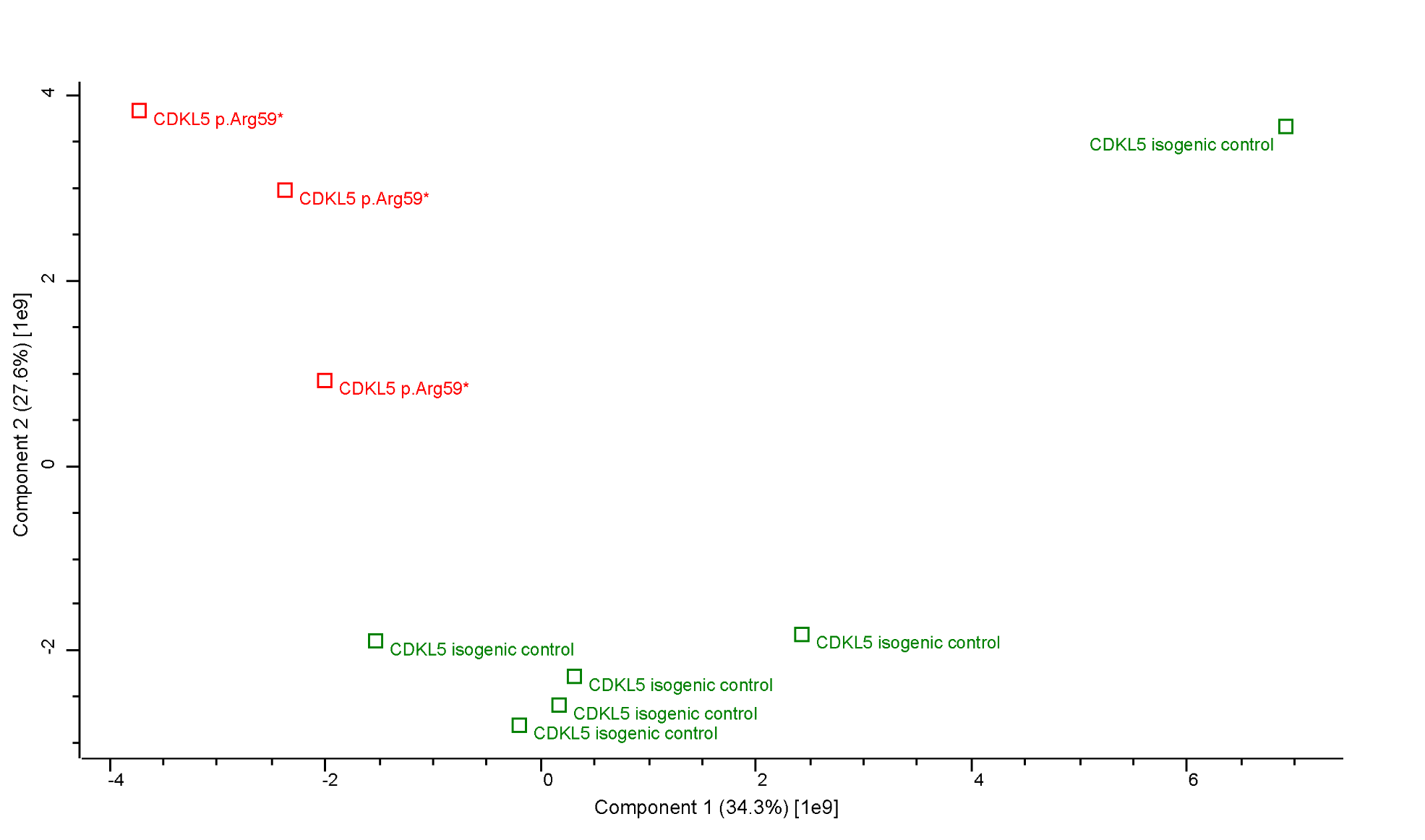
**

**Supplemental Fig. 1: Principal component analysis of phosphoproteomic data**

SP|Q8TAP8|PPR35_HUMAN P**RPDSP**QPRHGSPGRRKGRAERRGA-ARQRRQVRFRLTPPSPVRSEPQP---------AV 97

TR|A0A2K6B6K5|A0A2K6B6K5_MACNE P**RPDSP**ELRHGSPGRRKGRGEHRGT-ARQRRQVRFRLTPPSPVRSKPQP---------AV 96

TR|A0A2K5WZC8|A0A2K5WZC8_MACFA P**RPDSP**ELRHGSPGRRKGRGSIGAHPTALCSQVRFRLTPPSPVRSKPQP---------AV 97

TR|A0A6J3G0K2|A0A6J3G0K2_SAPAP P**RPDSP**EPRRGSPGRRKGRAERRGA-GRQRRQVRFRLAPPSPVRSEPQP---------AA 96

TR|A0A2K5PEK4|A0A2K5PEK4_CEBIM P**RPDSP**EPRRGSPGRRKGRAERRGA-GRQRRQVRFCLAPPSPVRSEPQP---------AA 96

TR|A0A2K6P3X8|A0A2K6P3X8_RHIRO L**RPDSP**EPRHCSPGRRKGRGVHRG-AARQRRQVRFRLTPPSPVRSKPQP---------AV 96

TR|H2QV27|H2QV27_PANTR P**RPDSP**QPRHGSPGRRKGRAERRGA-ARQRRQVRFRLTPPSPVRSEPQP---------AV 97

TR|G1RM09|G1RM09_NOMLE P**RPDSP**QPRHGSPGRRKGRAERRGE-ARQRRQVRFRLTPPSPVRSEPEP---------AL 96

TR|A0A2K6K2Y0|A0A2K6K2Y0_RHIBE L**RPDSP**EPRHCSPGRRKGRGVHRG-AARQRRQVRFRLTPPSPVRSKPQP---------AV 96

TR|A0A2K6V7D9|A0A2K6V7D9_SAIBB P**RPDSP**EPRPDSPGRRKGRAERRGA-GRQRRQVRFRLAPPSPVRSEPQP---------AA 96

TR|A0A2K5I7P1|A0A2K5I7P1_COLAP P**RPDSP**EPRHGSPGRRKGRGVHRG-AARQRRQVRFRLTPPSPVRSKPQP---------AV 96

TR|G3QRK9|G3QRK9_GORGO P**RPDSP**QPRHGSPGRRKGRAERRGA-ARQRRQVRFRLTPPSPVRSEPQP---------AV 97

TR|G7MNU4|G7MNU4_MACMU P**RPDSP**ELRHGSPGRRKGRGEHRGA-ARQRRQVRFRLTPPSPVRSKPQP---------AV 96

TR|F7ETS9|F7ETS9_MACMU P**RPDSP**ELRHGSPGRRKGRGEHRGA-ARQRRQVRFRLTPPSPVRSKPQP---------AV 96

TR|A0A6D2X5J5|A0A6D2X5J5_PANTR P**RPDSP**QPRHGSPGRRKGRAERRGA-ARQRRQVRFRLTPPSPVRSEPQP---------AV 97

TR|A0A2K5NWX7|A0A2K5NWX7_CERAT P**RPDSP**EPRHGSPGRRKGRGEHRGA-ARQRRQVRFRLTPPSPVRSKPQP---------AV 96

TR|A0A2J8RUX3|A0A2J8RUX3_PONAB P**RPDSP**QPRHGSPGRRKGRAERRGA-ARQRRQVRFRLTPPSPVRSEPQP---------AV 96

TR|A0A096N5S2|A0A096N5S2_PAPAN P**RPDSP**EPRHGSPGRRKGRGEHRGA-ARQRRQVRFRLTPPSPVRSKPQP---------AV 96

TR|A0A2I3HGB5|A0A2I3HGB5_NOMLE P**RPDSP**QPRHGSPGRRKGRAERRGE-ARQRRQVRFRLTPPSPVRSEPEP---------AL 97

TR|A0A0D9RZ77|A0A0D9RZ77_CHLSB P**RPDSP**EPRHGSPGRREHRGA-----ARQRRQVRFRLTPPSPVRSKPQP---------AV 92

TR|A0A2K5D7R2|A0A2K5D7R2_AOTNA P**RPDSP**EPRHGSPGRRKGRAERRSA-GRQRRQVRFRLAPPSPVRSEPQP---------AA 96

**Supplemental Fig. 2: Alignment of PPP1R35 demonstrates preservation of CDKL5 phosphomotif in primates**

**
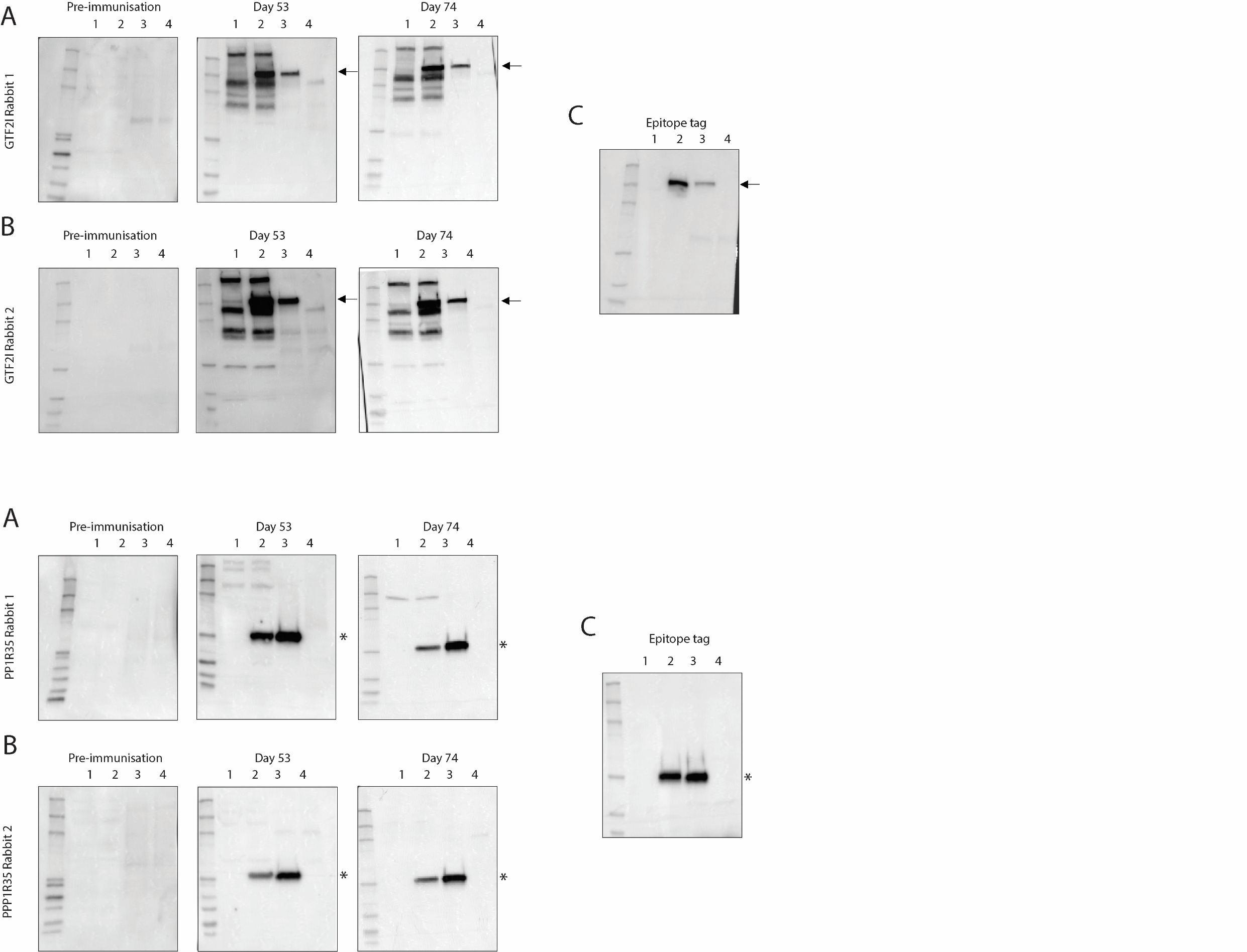
**

**Supplemental Fig. 3: Western blot testing of custom phospho antibodies PPP1R35-pS^52^**

Serum from rabbits was collected pre-immunisation and at days 53 and 74 for testing of immunoreactivity against expressed targets. HEK293T cells were either untransfected (lane 1) or transfected (lane 2) with PPP1R35-FLAG lysates harvested after 24-hours. Proteins from transfected cells were immunopurified with antibodies targeted against the FLAG epitope tag (lane 3) and were compared to parallel immunoprecipitations on untransfected HEK293T lysate (lane 4). Proteins were separated by SDS-PAGE, transferred to PVDF and then probed with serum. Serum from A) and B) two rabbits immunised against phosphor-PPP1R35 was tested for immunoreactivity against PPP1R35. As a positive control the same samples were run in parallel gels and tested for immunoreactivity against the FLAG epitope tag with α-FLAG antibody. A distinct band at the predicted molecular weight of PPP1R35-FLAG at ~29kDa (indicated with *) was detected in serum from days 53 and 74 in transfected HEK cell lysate (lane 2) and immunopurified PPP1R35-FLAG (lane 3) but was not detected in control samples (lanes 1 and 4). No bands were detected in serum collected pre-immunisation. C) Protein expression was confirmed with α-FLAG antibody.

**
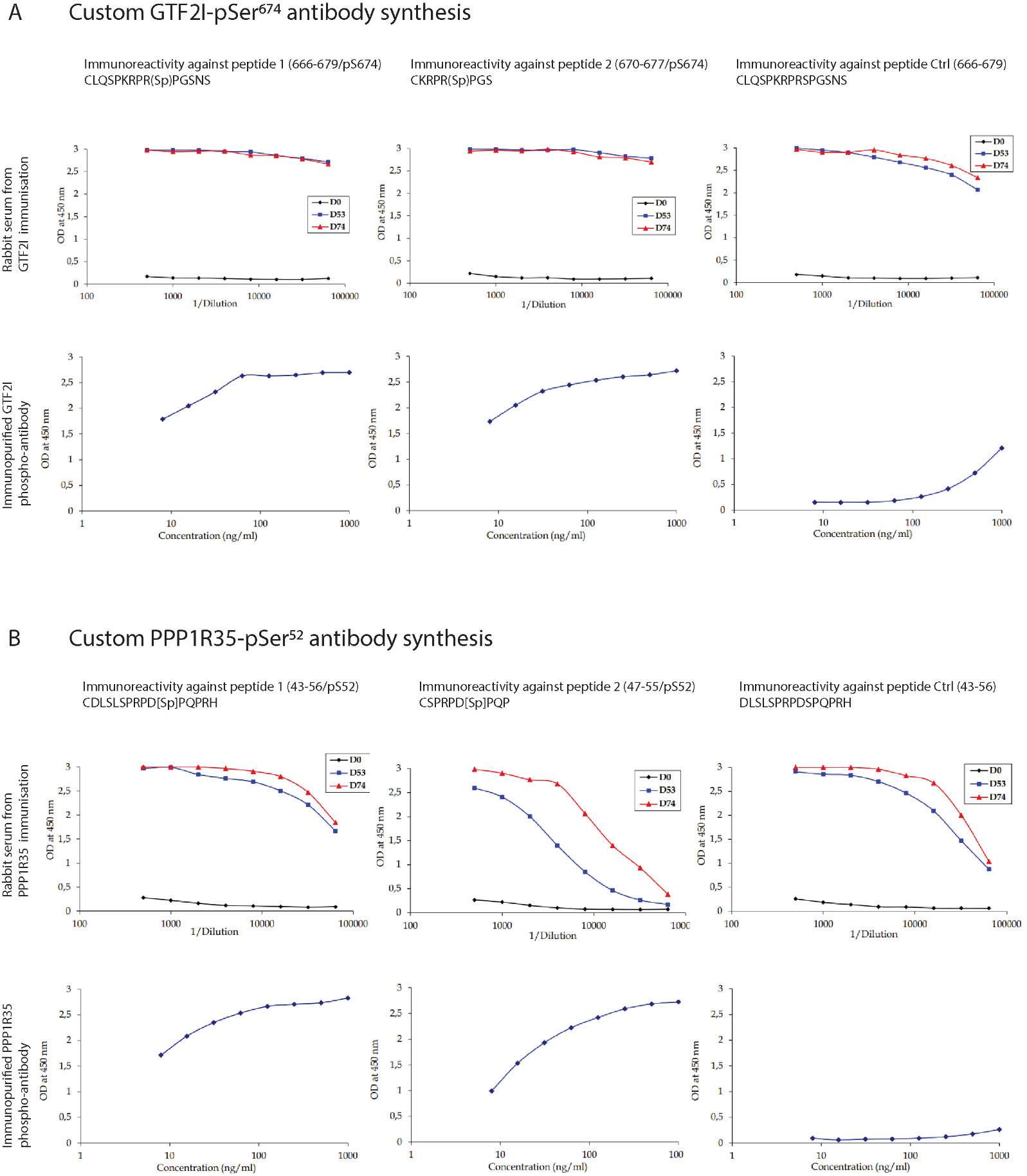
**

**Supplemental Fig. 4: ELISA testing of custom phospho-antibodies GTF2I-pS^674^ and PPP1R35-pS^52^**

Rabbits were immunised at days 0, 21, 42 and 53 using a 74-day protocol with three peptides for either GTF2I or PPP1R35 in Freund’s adjuvant. Two peptides contained the phospho-serine of interest, and one control peptide did not contain the phospho-serine which was used to induce a heightened immune reaction. Before immunisation and at days 53 and 74 test bleeds were taken and tested for immunoreactivity against all three peptides (top row of panels for each). Reactivity against each peptide increased over time. Antibodies against the phosphopeptides were then immunopurified on Sepahrose columns coupled to each respective Peptide 1 (containing a phospho-serine). Immunoreactivity of the antibodies against each of the phosphopeptides was tested by ELISA. For both GTF2I and PPP1R35 immunoreactivity against the two phosphopeptides was much higher than immunoreactivity against the control peptide (without the phosphoserine) indicating specificity against the phosphoserine in each target.


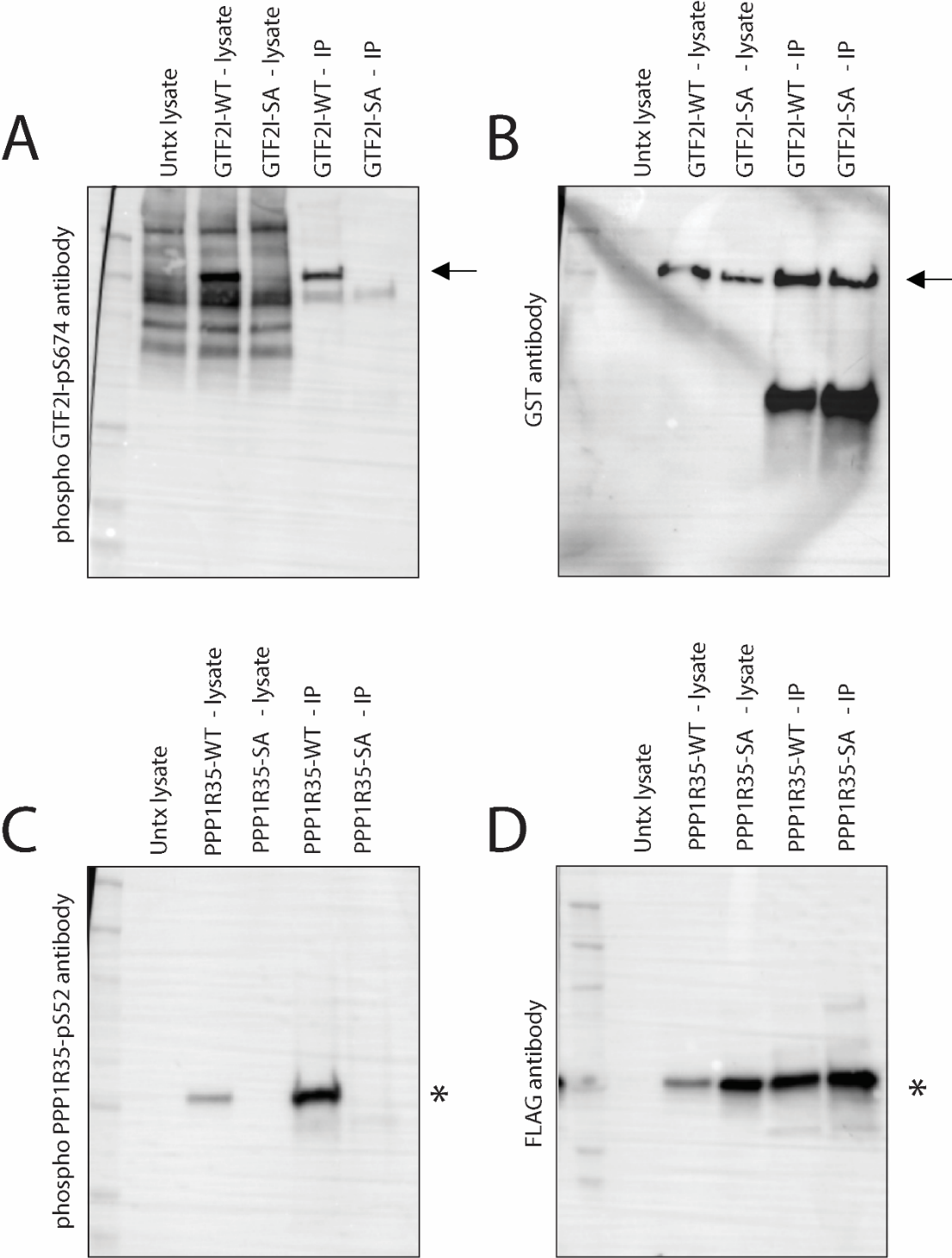


**Supplemental Fig. 5: Western blot testing of immunopurified custom phospho-antibodies for GTF2I-pS^674^ and PPP1R35-pS^52^**

A) Immunoreactivity of phospho-GTF2I antibody was tested against each of the target proteins by western blot. HEK293T cells were either untransfected (lane 1) or transfected with GTF2I WT (lane 2) or GTF2I S674A (lane 3) and lysates harvested after 24-hours. Proteins from cells were immunopurified with antibodies targeted against the GST epitope tag, and GTF2I WT immunoprecipitate (lane 4) compared to parallel immunoprecipitations of GTF2I S674A (lane 5). The phosphoantibody was specifically detecting GTF2I WT, not GTF2I S674A in both lysates (lane 2) and immunoprecipitated protein (lane 4).

B) As a control for transfection, parallel samples were probed with anti-GST to identify the epitope tag on GTF2I.

C) Immunoreactivity of phospho-PPP1R35 antibody was tested against each of the target proteins by western blot. HEK293 cells were either untransfected (lane 1) or transfected with PPP1R35 WT (lane 2) or PPP1R35 S52A (lane 3) and lysates harvested after 24-hours. Proteins from cells were immunopurified with antibodies targeted against the FLAG epitope tag, and PPP1R35 WT immunoprecipitate (lane 4) compared to parallel immunoprecipitations of PPP1R35 S52A (lane 5). The phosphoantibody was specifically detecting PPP1R35 WT, not PPP1R35 S52A in both lysates (lane 2) and immunoprecipitated protein (lane 4).

D) As a control for transfection, parallel samples were probed with anti-FLAG to identify the epitope tag on PPP1R35.

**
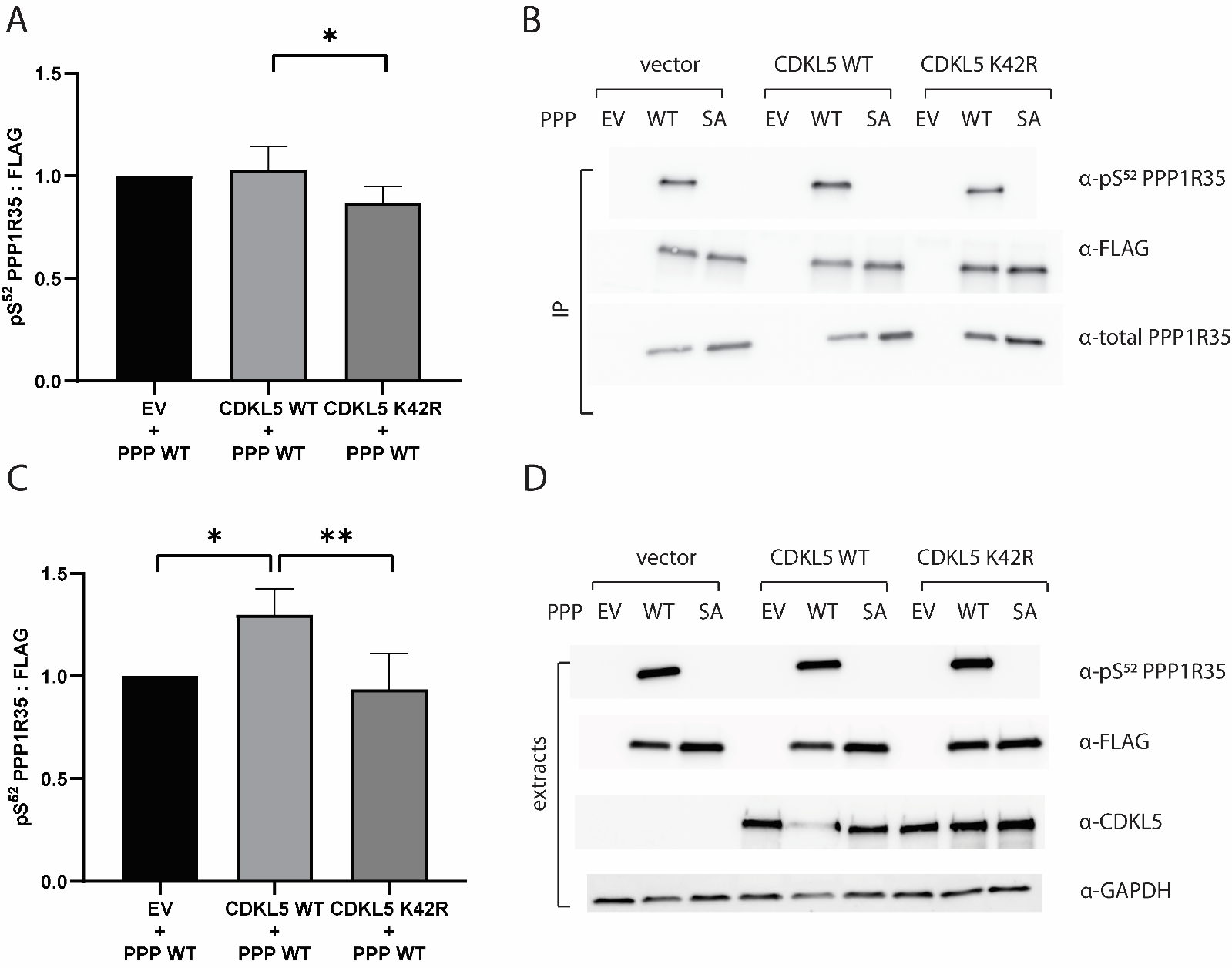
**

**Supplemental Fig. 6: Orthogonal validation approach to demonstrate that CDKL5 phosphorylates PPP1R35 at Ser^52^ in human cells using custom phospho-antibody**:

A) HEK293T cells were co-transfected with CDKL5 WT or CDKL5 kinase dead K42R and PPP1R35 WT or PPP1R35 S52A, where serine is converted to an alanine. Cells were then lysed and PPP1R35 was immunoprecipitated with the FLAG epitope tag. The membranes were probed with the custom pS^52^PPP1R35 antibody, FLAG antibody against the epitope tag, and total PPP1R35 antibody. Phosphorylation of PPP1R35 WT was higher in HEK293T cells co-transfected with CDKL5 WT compared to co-transfected with CDKL5 K42R when normalised to FLAG intensity. Data is mean +/- SEM. * = P<0.05. One-way ANOVA with Dunnett’s multiple comparisons test.

B) Representative western blot for results shown in A).

C) The lysate in A) was probed with the custom pS^52^PPP1R35 antibody, FLAG antibody against the epitope tag, total PPP1R35 and GAPDH. Reduced phosphorylation of Ser^52^ was confirmed in CDKL5 K42R compared to CDKL5 WT co-transfected with PPP1R35 WT.

D) Representative western blot for results shown in C)

Four independent experiments were done, and one representative experiment is shown. The expression patterns indicate that the co-transfection experiments were successful. Data is mean +/- SEM. * = P<0.05, ** = P<0.01. One-way ANOVA with Dunnett’s multiple comparisons test.

**
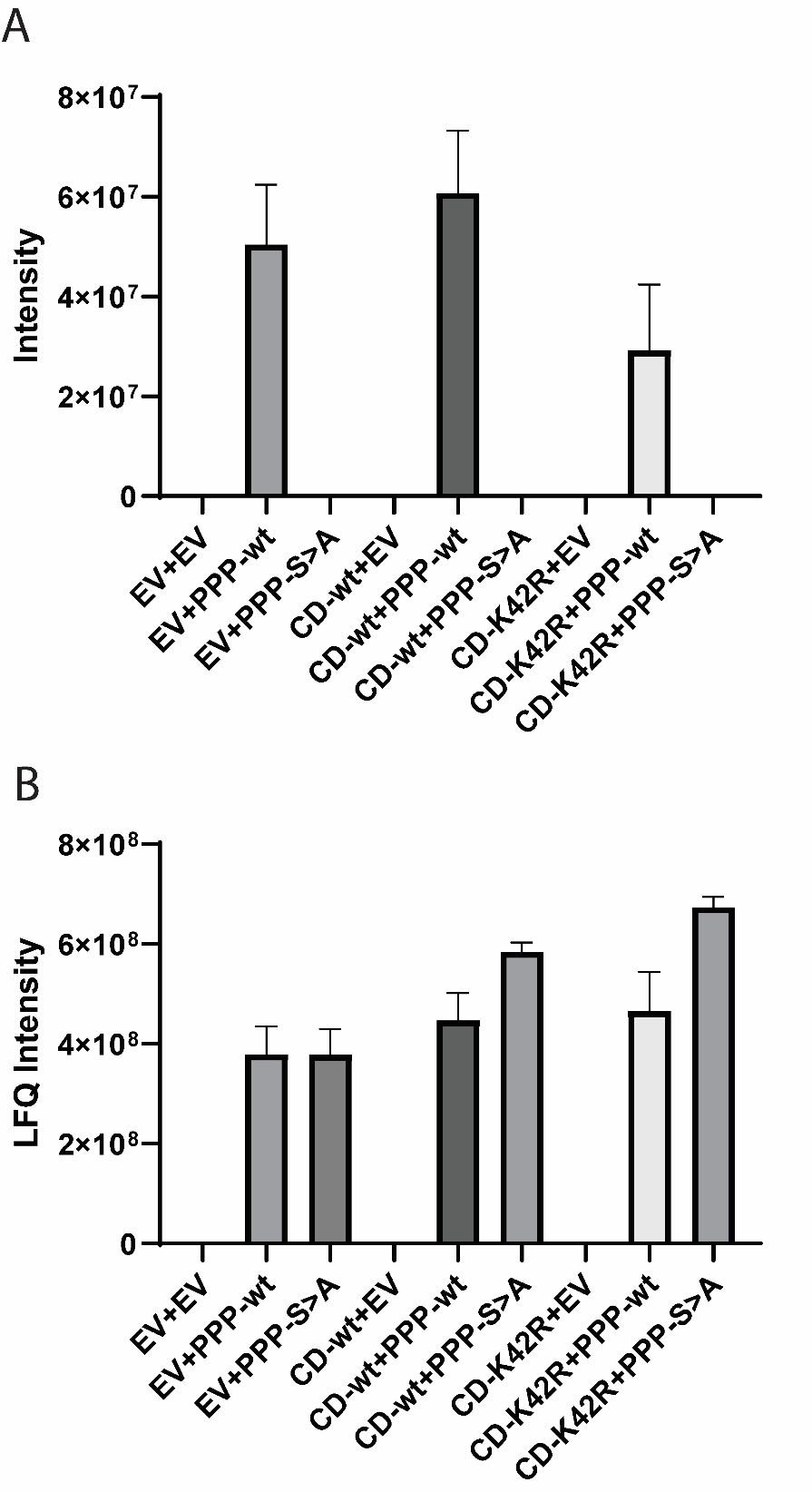
**

**Supplemental Fig. 7: Mass spectrometry analysis of PPP1R35 phosphorylation**

A) Raw phosphorylation levels of PPP1R35 at Ser^52^ and B) raw label-free phosphorylation levels of PPP1R35 total protein. Data is representative of 6 samples for PPP1R35SA and 10 independent samples of PPP1R35 WT from six independent experiments. CD; CDKL5, PPP; PPP1R35. Data is mean +/- SEM.


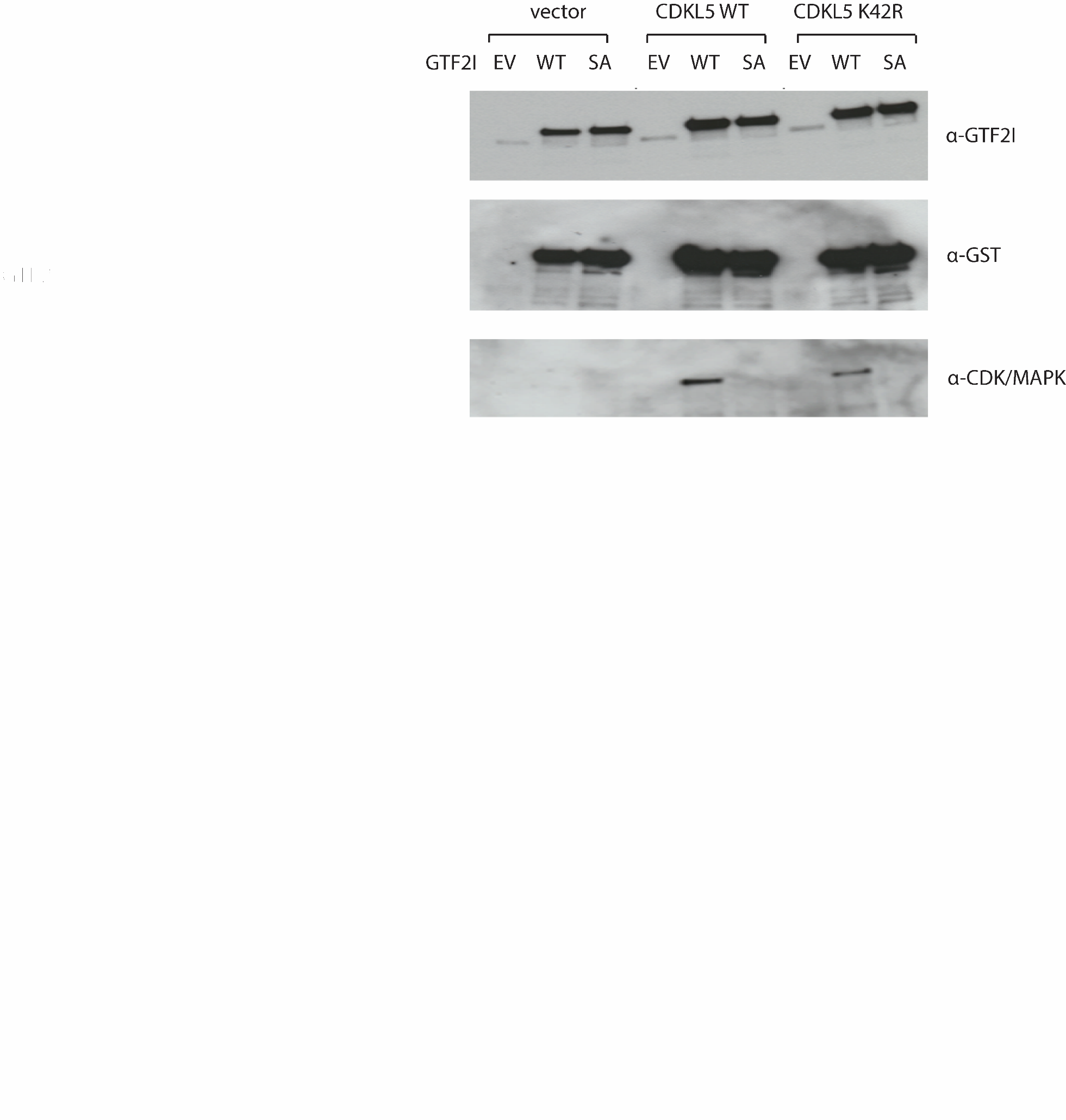


**Supplemental Fig. 8: Detection of phosphorylation of GTF2I using an antibody was used that detects phospho-MAPK/CDK substrates**

The antibody that detects phospho-MAPK/CDK substrates only worked reliably once in our experimental system in being able to detect phosphorylation in GTF2I as the antibody signal was quite faint. Highest phosphorylation was detected in HEK293 cells co-expressing CDKL5-WT and GTF2I-WT using the αCDK/MAPK antibody.

**
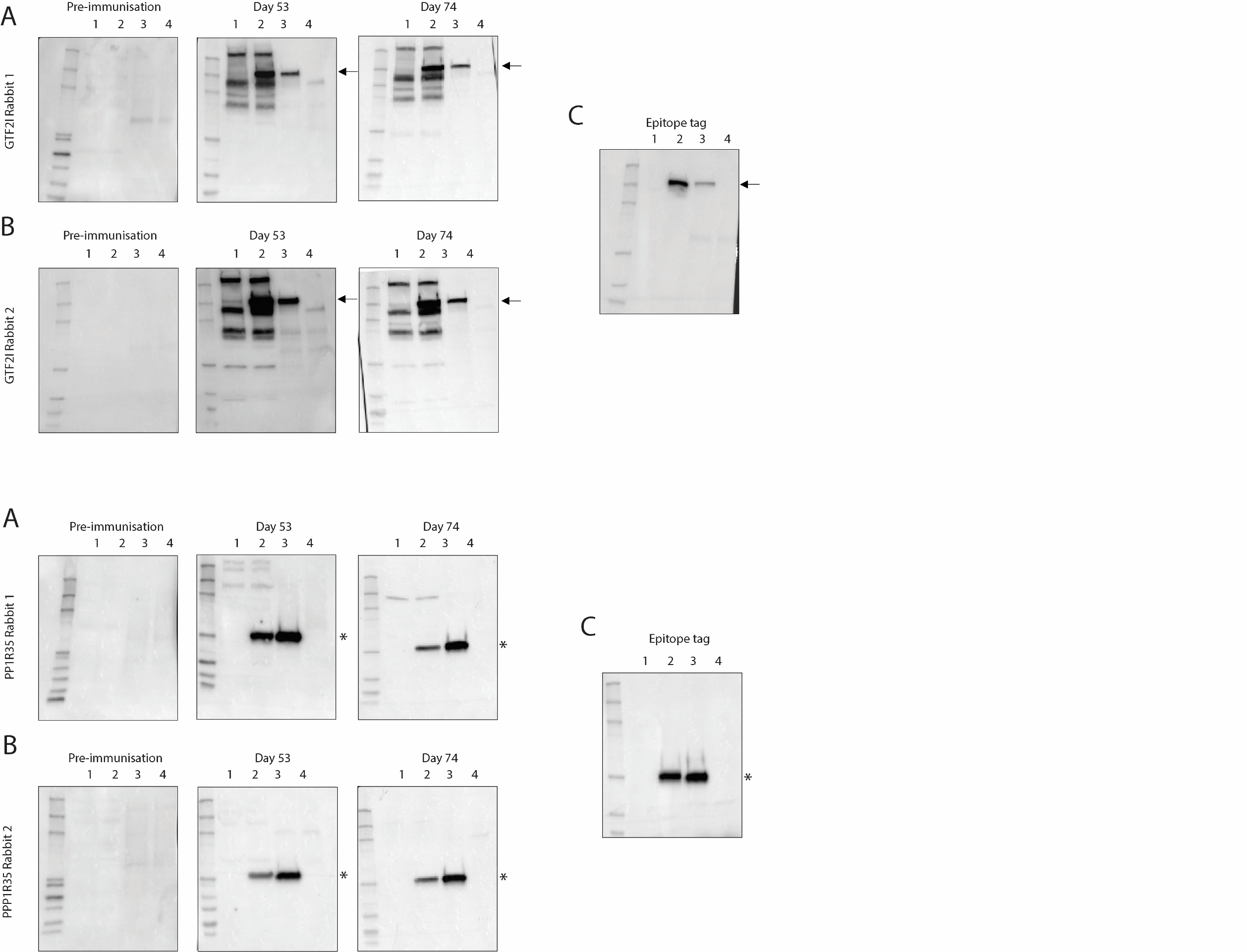
**

**Supplemental Fig. 9: Western blot testing of custom phospho-antibodies GTF2I-pS^674^**

Serum from rabbits was collected pre-immunisation and at days 53 and 74 for testing of immunoreactivity against expressed targets. HEK293 cells were either untransfected (lane 1) or transfected (lane 2) with GTF2I-GST lysates harvested after 24-hours. Proteins from transfected cells were immunopurified with antibodies targeted against the GST epitope tag (lane 3) and compared to parallel immunoprecipitations on untransfected HEK293T lysate (lane 4). Proteins were separated by SDS-PAGE, transferred to PVDF and then probed with serum. Serum from A) and B) two rabbits immunised against phosphor-GTF2I was tested for immunoreactivity against GTF2I. As a positive control the same samples were run on a parallel gel and tested for immunoreactivity against the GST epitope tag with α-GST antibody. A distinct band at the predicted molecular weight of GTF2I-GST at ~128kDa (indicated with an arrow) was detected in serum from days 53 and 74 in transfected HEK cell lysate (lane 3) and immunopurified GTF2I-GST (lane 4) but was not detected in control samples (lanes 1 and 4). No bands were detected in serum collected pre-immunisation. C) Protein expression was confirmed with α-GST antibody.

**
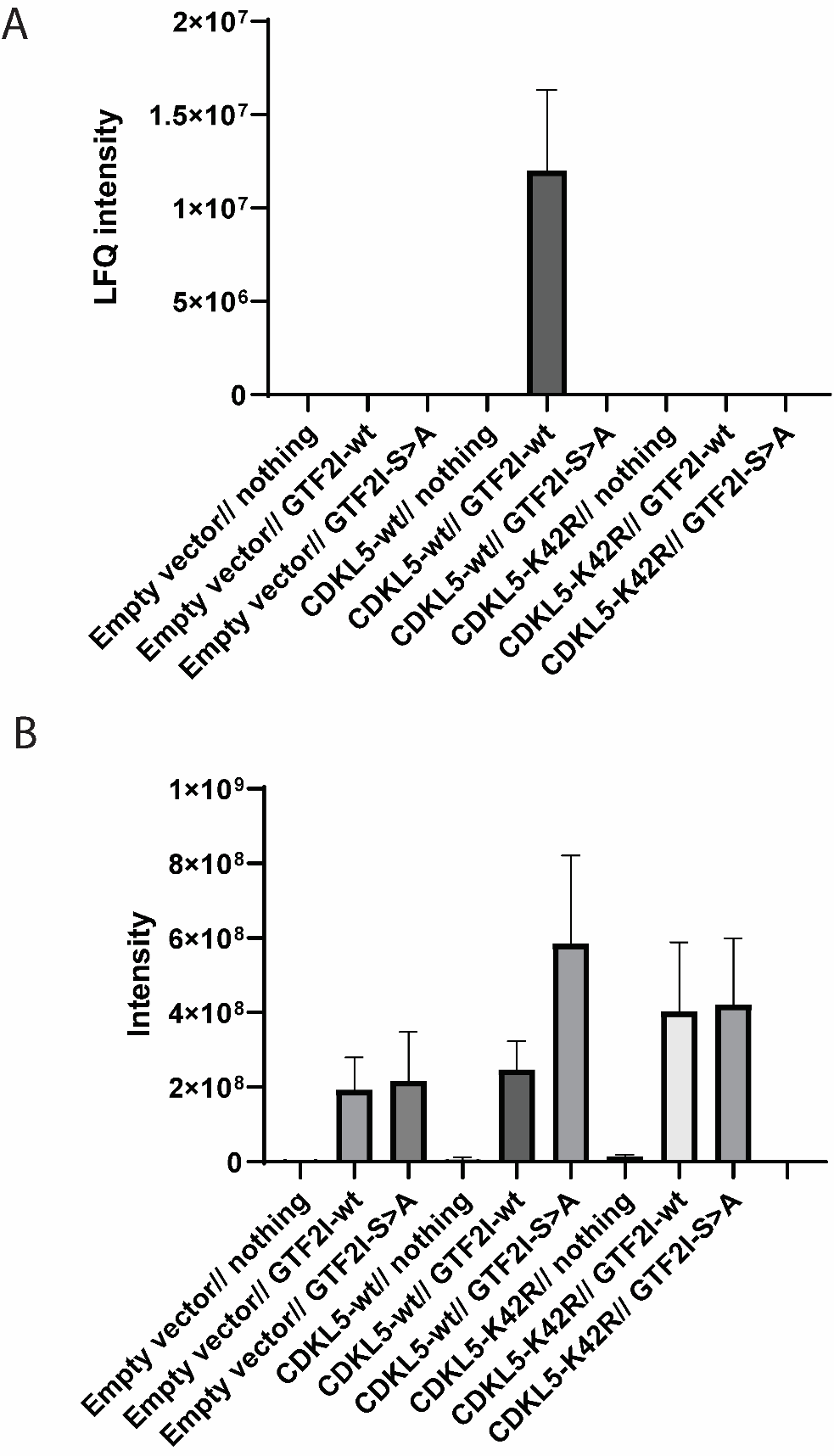
**

**Supplemental Fig. 10: Mass spectrometry analysis of GTF2I phosphorylation**

A) Raw phosphorylation levels of GTF2I at Ser^674^ was only detected in two experiments and was only ever detected when GTF2I WT was co-expressed with CDKL5 WT. B) Raw label-free phosphorylation levels of GTF2I total protein demonstrating loading of GTF2I into mass spectrometry experiments. Data is representative of 6 samples for total protein from six independent experiments. Data is mean +/- SEM.

**Supplemental Fig. 11: BEND4 expression in CDKL5 p.(Arg59*) and CDKL5 isogenic control neurons.**

There was a significant increase in expression (fold-change; 2^-(∆∆Ct)) of *BEND4* expression in CDKL5 p.(Arg59*) neurons compared to CDKL5 isogenic controls. ** = P<0.01. Data is mean +/- SEM. ** = P<0.01. Two-tailed Student’s t-test.

**
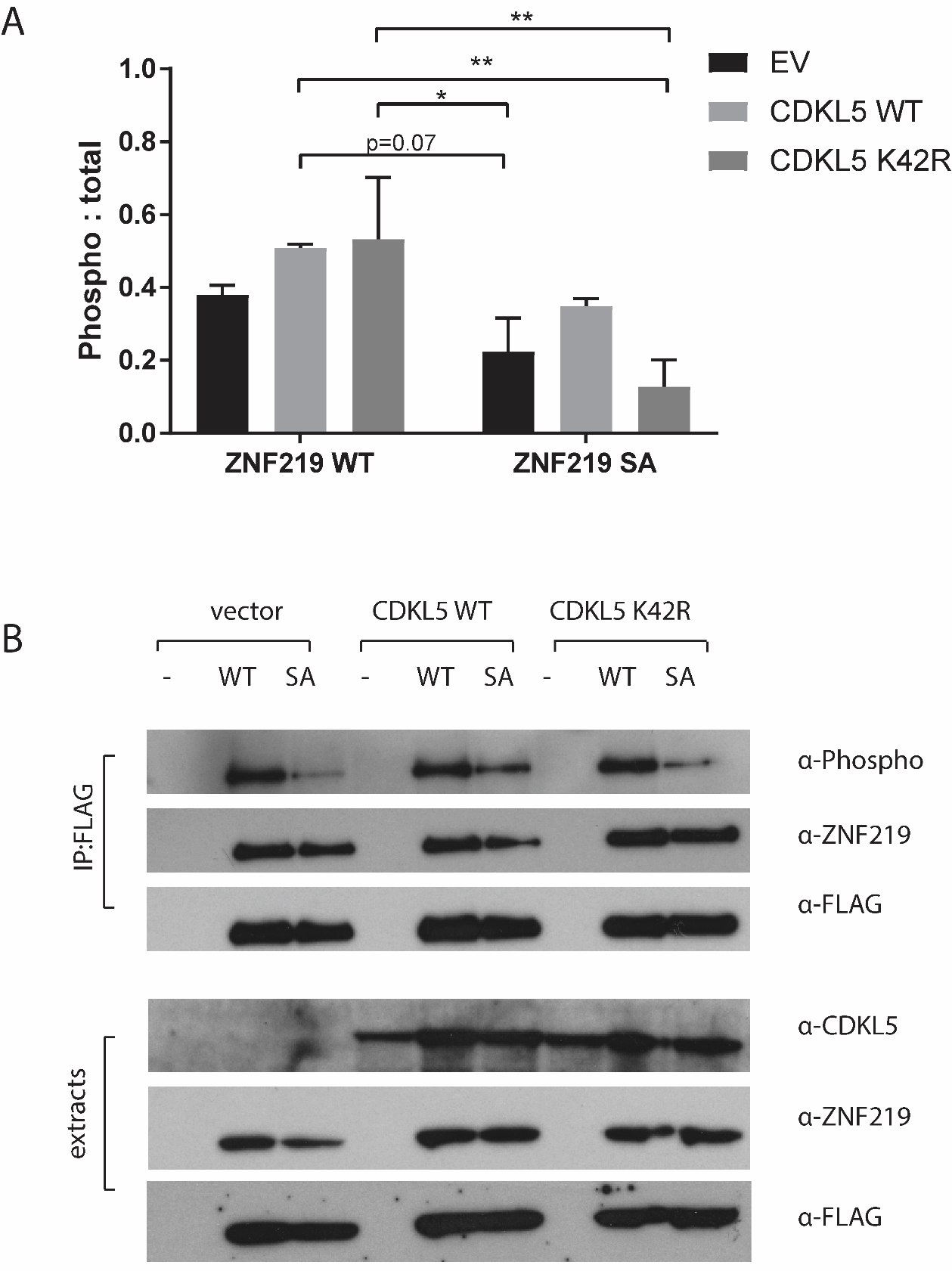
**

**Supplemental Fig. 12. CDKL5 does not phosphorylate ZNF219 at Serine114 in HEK293T cells**:

Anti‐FLAG immunoprecipitants (IP:FLAG) were probed with Anti-MAPK/CDK -Phospho) (top panel), anti-ZNF219 (middle panel) and anti-FLAG (bottom panel). Empty vector lysates (EV or “-“) were used as a negative control (-). Three independent experiments were done, and one representative experiment is shown. Quantification of three independent experiments showed no difference in the phosphorylation levels in ZNF219 WT, normalised to FLAG, when co-expressed with either empty vector, CDKL5 WT or CDKL5 KD (K42R) mutant. Site-directed mutagenesis did reduce overall phosphorylation levels when comparing ZNF219 WT ZN219 S114A (SA). Lysate (extract) sets from co-transfection experiments were probed with anti-CDKL5 (top panel), anti-ZNF219 (middle panel) and anti-FLAG (bottom panel). Five independent experiments were done, and one representative experiment is shown. The expression patterns indicate that the co-transfection experiments were successful. Data is mean +/- SEM. * = P<0.05, ** = P<0.01. One-way ANOVA with Sidak’s multiple comparisons test.

**
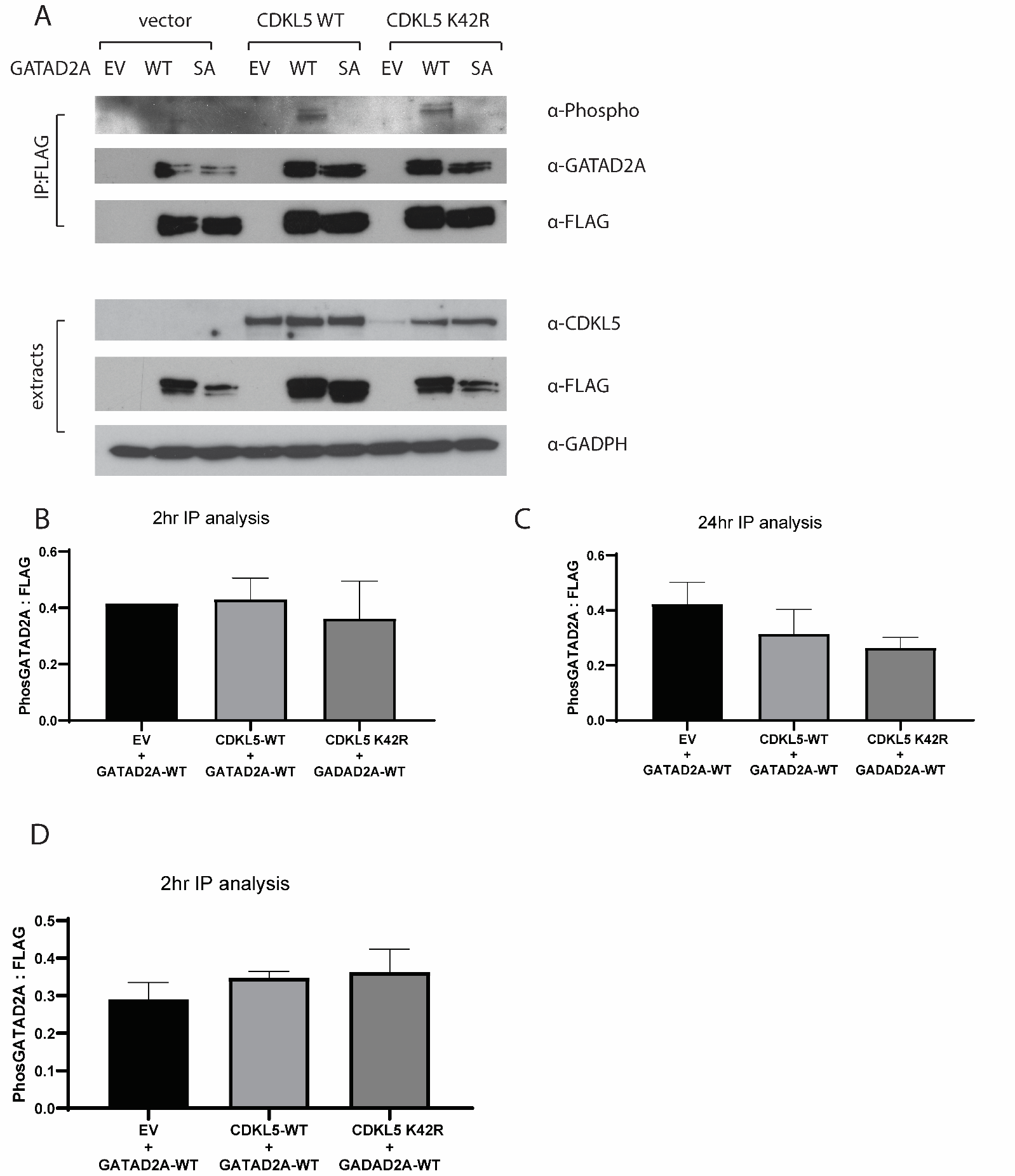
**

**Supplemental Fig. 13: CDKL5 does not phosphorylate GATAD2A at Serine100 in HEK293T cells**:

A) Anti‐FLAG immunoprecipitants (pulldown time of 2hr) were probed with Anti-MAPK/CDK -Phospho) (top panel), anti-GATAD2A (middle panel) and anti-FLAG (bottom panel). Empty vector lysates were used as a negative control (EV). Lysate sets from co-transfection experiments were probed with anti-CDKL5 (top panel), anti-FLAG (middle panel) and anti-GAPDH (bottom panel). Two independent experiments were done, and one representative experiment is shown. Site-directed mutagenesis completely abolished phosphosignal of GATAD2A S100A (SA).

B) Quantification of two independent experiments from 2hr pulldown time experiments showed no difference in the phosphorylation levels in GATAD2A WT when co-expressed with either empty vector, CDKL5 WT or CDKL5 KD (K42R) mutant. Note, the phosphosignal after 2hr pulldown experiments was faint, so extended pulldown time was tested.

C) Quantification of three independent experiments from 24hr pulldown time experiments showed no difference in the phosphorylation levels of GATAD2A WT when co-expressed with either empty vector, CDKL5 WT or CDKL5 KD (K42R) mutant.

D) Quantitative mass spectrometry of three independent experiments from 2hr pulldown IP samples showed no difference in phosphorylation levels of GATAD2A when co-expressed with either empty vector, CDKL5 WT or CDKL5 KD (K42R) mutant. Site-directed mutagenesis completely abolished phosphosignal of GATAD2A S100A.

. Data is mean +/- SEM.

**Supplemental Table 1: RNAseq data**

Large dataset uploaded as Excel file

| **Primer name** | **Forward primer** | **Reverse primer** |
| --- | --- | --- |
| GATAD2A cloning with FLAG tag | 5’-ACCAGGGATCCATGACCGAAGAAGCATGCCG-3’ | 5’-TGACTGCAAAGCGGCCGCTTTCCACGTGGCTGACTGGG-3’ |
| GATAD2A mutagenesis | 5'-atcacgtcaggtgcggggggtctcctc-3' | 5'-gaggagaccccccgcacctgacgtgat-3' |
| GTF2I cloning with FLAG tag | 5’-ACCAGGGATCCATGGCCCAAGTTGCAATGTC-3’ | 5’-TGACTGCAAAGCGGCCGCCCACGTGGGGTCTGGTTCT-3’ |
| GTF2I mutagenesis | 5'-ttactcccaggagctcgtggtcttttgggggactgc-3' | 5'-gcagtcccccaaaagaccacgagctcctgggagtaa-3' |
| ZNF219 cloning with FLAG tag | 5’-ACCAGGGATCC ATGGAGGGCTCACGTCCCCG-3’ | 5’-TGACTGCAAAGCGGCCGCCCGTTCTTGCCCCCCCAGCC-3’ |
| ZNF219 mutagenesis | 5'-cgtgcagcaggagcacgtgggcgctcggg-3' | 5'-cccgagcgcccacgtgctcctgctgcacg-3' |
| PPP1R35 cloning with FLAG tag | 5’-ACCAGGGATCCATGATGATGGGTTGTGGGGA-3’ | 5’-TGACTGCAAAGCGGCCGCCGCTTCCCATCGCCTCAGT-3’ |
| PPP1R35 mutagenesis | 5'-cgcggcccgacgcccctcagccgc-3' | 5'-gcggctgaggggcgtcgggccgcg-3' |
| BEND4 qPCR | 5'-GGCTATTGTCCTCGATGCCAAG-3' | 5'-TCAGGAGTTGGTTGTGGTGCTG-3' |
| GAPDH qPCR | 5'-CATGAGAAGTATGACAACAGCCT-3' | 5'-AGTCCTTCCACGATACCAAAGT-3' |
| B-actin qPCR | 5'-ACCATGGATGATGATATCGC-3' | 5'-TCATTGTAGAAGGTGTGGTG-3' |
| HPRT1 qPCR | 5'-AGGCGAACCTCTCGGCTTTC-3' | 5'-CTGGTTCATCATCACTAATCACGAC-3' |
| TBP qPCR | 5'-GCAAGGGTTTCTGGTTTGCC-3' | 5'-CAAGCCCTGAGCGTAAGGTG-3' |

**Supplemental Table 2: Primers**

| **Target** | **Catalogue number** | **Supplier** | **Dilution** | **Blocking buffer** |
| --- | --- | --- | --- | --- |
| Phospho-MAPK/CDK Substrates (PXS*P or S*PXR/K) | 2325S | Cell Signaling | 1:1000 | 5 % Milk in TBST (0.1%) |
| Phospho GTF2I S^674^ | Custom synthesis | Covalab | 0.5ug/ml | 5% Milk in TBST (0.1%) |
| Phospho PPP1R35 S^52^ | Custom synthesis | Covalab | 0.5ug/ml | 5% Milk in TBST (0.1%) |
| FLAG | F1804-1MG | Sigma-Aldrich | 1:2000 | 5% Milk in TBST (0.1%) |
| GST | 2622S | Cell Signaling | 1:2000 | 5% Milk in TBST (0.1%) |
| CDKL5 | S957D | MRC PPU | 1 ug/ml | 5% Milk in TBST (0.1%) |
| GAPDH | G9545 | Sigma Aldrich | 1:5000 | 5% Milk in TBST (0.1%) |
| PPP1R35 | TA334819 | Origene | 1:2000 | 5% Milk in TBST (0.1%) |
| GTF2I | HPA026638 | Sigma Aldrich | 1:2000 | 5% Milk in TBST (0.1%) |
| ZNF219 | HPA056168 | Sigma Aldrich | 1:2000 | 5% Milk in TBST (0.1%) |
| GATAD2A | ab87663 | Abcam | 1:2000 | 5% Milk in TBST (0.1%) |
| Anti-Rabbit HRP | 7074S | Cell Signaling | 1:5000 | 5% Milk in TBST (0.1%) |
| Anti-Mouse HRP | NA931VS | Bio-Strategy | 1:5000 | 5% Milk in TBST (0.1%) |
| Anti-Sheep HRP | ab6747 | Abcam | 1:5000 | 5% Milk in TBST (0.1%) |

**Supplemental Table 3: Antibodies and dilutions**

**Supplemental Table 4: MaxQuant search results from global phosphoproteomic data of CDKL5 isogenic and p.Arg59* neurons**

Raw intensity files from MaxQuant search prior to Perseus data analysis. Large dataset uploaded as Excel file

| **Uniprot ID** | **Protein** | **Amino acid** | **Position** | **PhosphoSitePlus kinase/s** | **Human CDKL5 p.Arg59* neurons** |
| --- | --- | --- | --- | --- | --- |
| F6S8Q4 | DDX3X | S | 561 | AurB | ↓ |
| P20700 | LMNB1 | S | 23 | CDK1 | ↓ |
| P20700 | LMNB1;LMNB2 | S | 393 | CDK1 | ↓ |
| Q9ULW0 | TPX2 | S | 738 | CDK1 | ↓ |
| F2Z357 | RAP1GAP | S | 484 | CDK1 | ↑ |
| P19338 | NCL | T | 121 | CDK1;CDC7;CDK2 | ↓ |
| P19338 | NCL | T | 121 | CDK1;CDC7;CDK2 | ↓ |
| Q9H1E3 | NUCKS1 | S | 181 | CDK1;CDK2 | ↓ |
| A0A286YFD6 | RRM2 | S | 20 | CDK1;CDK2 | ↓ |
| Q03252 | LMNB2 | S | 37 | CDK2 | ↓ |
| Q13242 | SRSF9 | S | 216 | CDK2 | ↓ |
| Q9C0C2 | TNKS1BP1 | S | 691 | CDK2 | ↓ |
| Q14194-2 | CRMP1 | T | 623 | CDK2;CDK5 | ↑ |
| P12931 | SRC | S | 75 | CDK5 | ↑ |
| Q14195-2 | DPYSL3 | S | 636 | CDK5;DYRK2 | ↑ |
| P17096-1 | HMGA1 | S | 102 | CK2A1 | ↓ |
| P11388-1 | TOP2A | S | 1525 | CK2A1;CDC7;PLK1 | ↓ |
| P49736 | MCM2 | S | 13 | CK2A1;CDK7;CDK2 | ↓ |
| P16104 | H2AFX | T | 137 | DNAPK | ↓ |
| P08670 | VIM | S | 459 | DNAPK;MOS | ↓ |
| P16104 | H2AFX | S | 140 | DNAPK;MST1 | ↓ |
| Q96S94-3 | CCNL2 | S | 116 | DYRK1A | ↓ |
| A0A2R8Y5G2 | EPB41 | Y | 660 | EGFR | ↓ |
| P11388-1 | TOP2A | S | 1247 | ERK1;CDK1;ERK2 | ↓ |
| P78347-2 | GTF2I | S | 627 | ERK1;ERK2 | ↓ |
| P78347-2 | GTF2I | S | 633 | ERK1;ERK2 | ↓ |
| P78347-2 | GTF2I | S | 627 | ERK1;ERK2 | ↓ |
| I3L170 | MAPT | T | 148 | ERK1;GSK3B;ERK2;DYRK1A;CDK5;JNK2;P38D | ↑ |
| B3KR49 | MAPK3 | Y | 90 | ERK1;JAK2;Lck;MEK1;MEK2;Ret | ↑ |
| Q9UIG0-2 | BAZ1B | S | 158 | ERK1;P38A;JNK2 | ↓ |
| I3L170 | MAPT | S | 300 | GSK3A;GSK3B | ↑ |
| I3L170 | MAPT | S | 130 | GSK3A;PKACA;ERK1;GSK3B;ERK2;DYRK1A;CDK5;TTBK1 | ↑ |
| I3L170 | MAPT | S | 296 | GSK3A;PKCA;CAMK2A;AMPKA1;ERK1;CK1D;MARK1;GSK3B;P38G;ERK2;DYRK1A;CDK5;P38D;JNK1 | ↑ |
| I3L170 | MAPT | S | 296 | GSK3A;PKCA;CAMK2A;AMPKA1;ERK1;CK1D;MARK1;GSK3B;P38G;ERK2;DYRK1A;CDK5;P38D;JNK1 | ↑ |
| I3L170 | MAPT | S | 296 | GSK3A;PKCA;CAMK2A;AMPKA1;ERK1;CK1D;MARK1;GSK3B;P38G;ERK2;DYRK1A;CDK5;P38D;JNK1 | ↑ |
| I3L170 | MAPT | T | 162 | GSK3A;PKCA;CAMK2A;PKACA;AMPKA1;ERK1;CDK1;MARK1;GSK3B;ERK2;CDK5;DYRK1A;JNK2;JNK1 | ↑ |
| I3L170 | MAPT | S | 166 | GSK3A;PKCA;CAMK2A;PKACA;ERK1;CDK1;GSK3B;ERK2;CDK5 | ↑ |
| I3L2R2 | FAM64A | S | 23 | KIS | ↓ |
| P46937-5 | YAP1 | S | 109 | LATS1;NDR1;PKCZ | ↓ |
| F8WE04 | HSPB1 | S | 78 | MAPKAPK2;PKACA;P70S6KB;PKG1 iso2 | ↓ |
| F8WE04 | HSPB1 | S | 82 | MAPKAPK2;PKACA;P70S6KB;PKG1 iso2;PRKD1;Akt1 | ↓ |
| B3KR49 | MAPK3 | T | 88 | MEK1;MEK2 | ↑ |
| Q53EL6-2 | PDCD4 | S | 446 | p90RSK;Akt1 | ↓ |
| O60885-1 | BRD4;PDHA1;OR2C1;SESN3 | S | 1739 | PDK1;PDHK2;PDHK1 | ↓ |
| P08670 | VIM | S | 39 | PKACA;PAK1;Akt1 | ↑ |
| Q9NPB6-2 | PARD6A | S | 344 | PKCI | ↑ |
| K7ELV5 | CDK3;CDK2 | T | 14 | Wee1 | ↓ |
| K7ELV5 | CDK3;CDK2 | Y | 15 | Wee1 | ↓ |

**Supplemental Table 5: Significantly altered phosphosites from phosphoproteomic dataset that have reported kinases**

Of the 454 significantly altered phosphosites between CDKL5 p.(Arg59*) and CDKL5 isogenic controls, 48 sites had a reported kinase in PhosphoSitePlus.

| **Motif and frequency** | **Site** | **Score** | **fg_ match** | **fg_ size** | **bg_ match** | **bg_ size** | **Fold-enrichment** | **unadjusted_ p-value** | **tests** | **adjusted_ p-value** |
| --- | --- | --- | --- | --- | --- | --- | --- | --- | --- | --- |
| \| 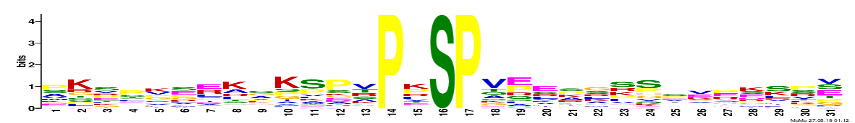 \| \| --- \| | S | 78.77 | 27 | 191 | 1 | 191 | 27 | 4.30E-08 | 107 | 4.60E-06 |
| 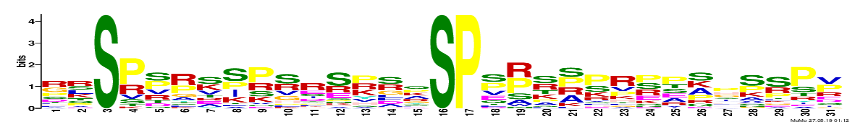   \|  \| \| --- \| | S | 57.18 | 26 | 191 | 1 | 191 | 26 | 8.90E-08 | 71 | 6.40E-06 |
| 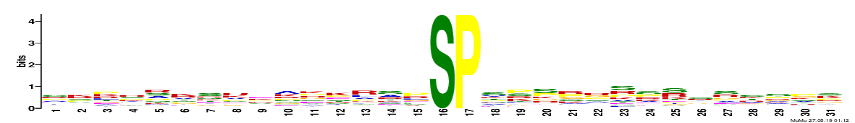 | S | 35.3 | 119 | 191 | 19 | 191 | 6.3 | 2.90E-28 | 28 | 8.20E-27 |
| 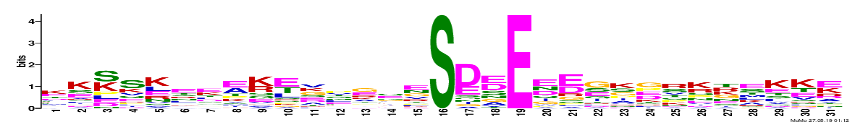   \|  \| \| --- \| | S | 7.18 | 30 | 191 | 12 | 191 | 2.5 | 2.50E-03 | 3 | 7.40E-03 |
| 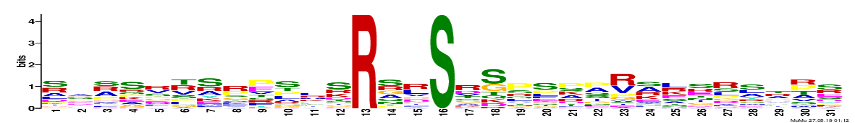   \|  \| \| --- \| | S | 7.17 | 49 | 191 | 18 | 191 | 2.7 | 2.10E-05 | 1 | 2.10E-05 |
| **Combined common motif** |  |  |  |  |  |  |  |  |  |  |
| 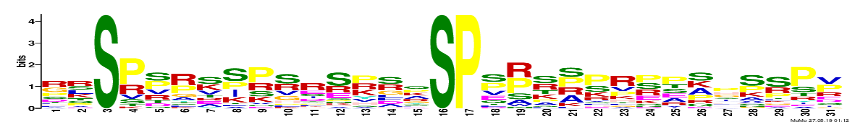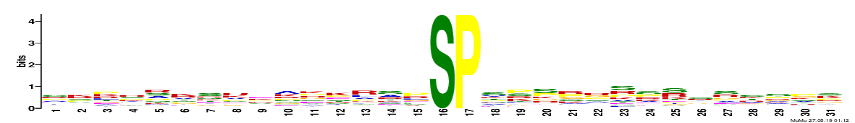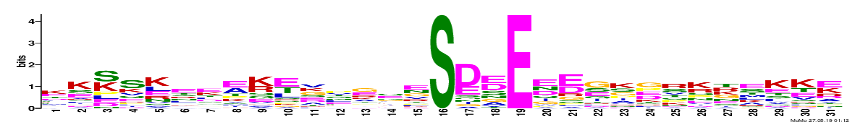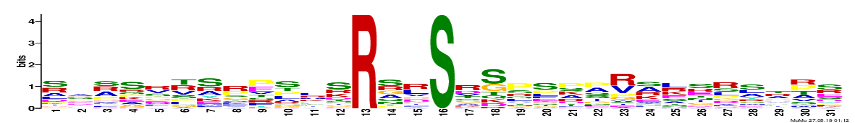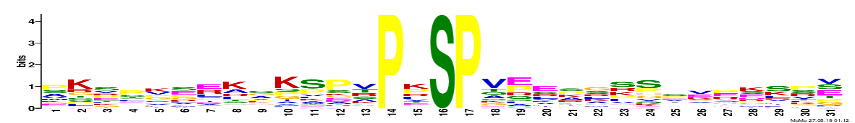  \|  \| \| --- \| | S |  |  |  |  |  |  |  |  |  |

**Supplemental Table 6: Motif enrichment in phosphoproteomics data from CDKL5 human neurons**

Significantly altered phosphopeptides were uploaded into MoMo for matrix enrichment analysis. PxS_p_P was the most highly enriched phosphotmotif in the dataset. The consensus motif RPxS_p_PxE closely resembled the published CDKL5 phosphomotif RPX(S_p_/T_p_[]A/G/P]. *fg_match*: is the number of foreground peptides that match the motif. Note: For motif-x, the foreground counts are based on all the peptides unless the --harvard option is specified, in which case the counts are based on the remaining peptides. *fg_size*: is the total number of foreground peptides with the given central modification. *bg_match*: is the number of background peptides that match the motif. Note: For motif-x, the background counts are based on all the peptides unless the --harvard option is specified, in which case the counts are based on the remaining peptides. *bg_size*: is the total number of background peptides with the given central modification The fold enrichment is of the foreground matches vs. the background matches. This is equal to:  (fg_match / fg_size) / (bg_match / bg_size). *Unadjusted p-value*: The p-value of the Fisher Exact test on the enrichment of the motif in the foreground vs. the background peptides. This value does not accurately represent the statistical significance of the motif, and should be interpreted as a score only.

| Protein | Site of phosphorylation | Peptide identified by phosphor-mass spectrometry | PEP | Site localization probability | Score | -Log Student's T-test p-value Mut_  WT | Student's T-test Difference Mut_WT | Present in databases  ^#^ | Potential upstream regulatory protein that targets that particular phosphosite ^#^ | Potential treatment that targets that particular phosphosite ^#^ | Conserved (in mouse and rat)? | Ranked likelihood CDKL5 phosphorylating this site ^$^ | Site percentile ^$^ | Log_2_ score ^$^ |
| --- | --- | --- | --- | --- | --- | --- | --- | --- | --- | --- | --- | --- | --- | --- |
| GATAD2A | S100-p | **RPPSP**DVIVLSDNEQPSSPR | 6.37E-28 | 1.000000 | 162.15 | 5.4003 | -1.9427 | 1 LTP  110 HTP | TBK1  Can be phosphorylated by AMPK | dasatinib | Yes | 14^th^ | 99.578 | 5.715 |
| GTF2I | S674-p | ALQSPK**RPRSP**GSNSK | 4.85E-05 | 1.000000 | 109.66 | 4.4320 | -2.1272 | 1 LTP  52 HTP | No. | None known | Yes | 6^th^ | 99.935 | 7.372 |
|  |  | **RPRSP**GSNSKVPEIEVTVEGPNNNNPQTSAVR | 1.75E-196 | 0.998962 | 263.38 | 3.2731 | -2.1313 |  |  |  |  |  |  |  |
| ZNF219 | S114-p | THQPE**RPRSP**AAR | 4.05E-06 | 0.999992 | 80.738 | 2.2726 | -1.3793 | 0 LTP  33 HTP | No | None known | Yes | 10^th^ | 99.805 | 6.611 |
| PPP1R35 | S52-p | APVPEPGLDLSLSP**RPDSP**QPR | 2.93E-24 | 0.999788 | 143.05 | 2.6434 | -1.0738 | 0 LTP  48 HTP | No. | BI2536  MLN8054  nocodazole | No | 2^nd^ | 99.848 | 6.801 |

**Supplemental Table 7: Phosphoproteins with CDKL5 motif identified in mass spectrometry analysis of CDKL5 in human neurons**

Peptides identified in phosphoproteomic dataset representing potential CDKL5 targets. GTF2I was identified by two independent peptides mapping to the same phosphorylation sites.

^#^,checked in Phosphosite.org ^1^, LTP = low throughput content, HTP = high throughput content; $, checked in Kinase Library within Phosphosite Plus ^2^

**Supplemental Table 8: MaxQuant search results from PPP1R35 immunoprecipitation mass spectrometry analysis**

Raw intensity files from MaxQuant search prior to Perseus data analysis. Large dataset uploaded as Excel file

**Supplemental Table 9: MaxQuant search results from GTF2I immunoprecipitation mass spectrometry analysis**

Raw intensity files from MaxQuant search prior to Perseus data analysis. Large dataset uploaded as Excel file

| **Model system** | **Citation** | **Human Gene** | **Effect in model system** | **Human CDKL5 p.Arg59* neurons** | **RNAseq data** | | | **qPCR data** | |
| --- | --- | --- | --- | --- | --- | --- | --- | --- | --- |
|  |  |  |  |  | **logFC#** | **logCPM** | **FDR** | **FC*** | **P-value** |
| Human WS iPSC | Adamo *et al.* 2015 | *BEND4* | ↑ | ↑ | 2.62 | 3.61 | 3.06E-02 | 19.04 | 2.15E-03 |
| Human WS fibroblasts | Henrichsen et al 2011 | *CYP26B1* | ↑ | ↑ | 3.47 | 5.52 | 3.83E-02 | NM | NM |
|  |  | *PID1* | ↑ | ↑ | 2.43 | 4.53 | 9.34E-04 | NM | NM |
|  |  | *SPON1* | ↑ | ↑ | 4.35 | 5.03 | 6.37E-04 | NM | NM |
|  |  | *COL5A2* | ↓ | ↓ | -2.70 | 6.64 | 2.01E-03 | NM | NM |
|  |  | *CYP1B1* | ↓ | ↓ | -5.04 | 6.89 | 4.64E-03 | NM | NM |
|  |  | *RARRES2* | ↓ | ↓ | -4.40 | 4.38 | 1.24E-11 | NM | NM |
|  |  | *SLC39A8* | ↓ | ↓ | -3.49 | 2.52 | 3.84E-03 | NM | NM |
| Human WS post-mortem brain biopsy | Barak *et al.* 2019 | *COL4A5* | ↓ | ↓ | -2.78 | 6.48 | 1.17E-04 | NM | NM |
|  |  | *COL9A3* | ↓ | ↓ | -2.65 | 2.43 | 3.45E-02 | NM | NM |
|  |  | *ERBB3* | ↓ | ↓ | -5.24 | 2.37 | 6.23E-03 | NM | NM |
|  |  | *MAL* | ↓ | ↓ | -2.47 | 0.40 | 3.45E-02 | NM | NM |
|  |  | *PCSK6* | ↓ | ↓ | -3.03 | 2.50 | 8.14E-03 | NM | NM |
|  |  | *SEMA3B* | ↓ | ↓ | -2.97 | 2.74 | 5.86E-03 | NM | NM |
|  |  | *SEPP1* | ↓ | ↓ | -2.24 | 4.63 | 4.94E-02 | NM | NM |
|  |  | *SMIM5* | ↓ | ↓ | -7.77 | 0.57 | 6.23E-03 | NM | NM |
|  |  | *TPPP3* | ↓ | ↓ | -5.25 | 5.25 | 7.17E-09 | NM | NM |
|  |  | *VRK2* | ↓ | ↓ | -4.53 | 3.42 | 4.47E-05 | NM | NM |
|  |  | *LPPR1* | ↓ | ↑ | 2.01 | 5.70 | 4.79E-02 | NM | NM |
|  |  | *PLP1* | ↓ | ↑ | 2.28 | 6.78 | 2.48E-02 | NM | NM |
| *Gtf2i* knockout mouse cortex |  | *DLK1* | ↓ | ↓ | -3.25 | 6.04 | 1.16E-04 | 0.17 | 8.23E-04 |
|  |  | *MAL* | ↓ | ↓ | -2.47 | 0.40 | 3.45E-02 | NM | NM |
|  |  | *PLP1* | ↓ | ↑ | 2.28 | 6.78 | 2.48E-02 | 4.72 | 2.46E-03 |
|  |  | *GJB7* | ↓ mouse *Gjc2* | ↓ | -6.86 | -0.19 | 1.73E-02 | NM | NM |
|  |  | *GJA1* |  | ↓ | -2.05 | 5.65 | 7.87E-02 | 0.35 | 3.77E-03 |
|  |  | *CLDN6* | ↓ mouse *Cldn11* | ↓ | -3.68 | 1.81 | 1.79E-02 | NM | NM |
|  |  | *CLDN1* |  | ↓ | -1.83 | 3.98 | 2.53E-02 | NM | NM |
|  |  | *CLDN4* |  | ↓ | -3.30 | 0.38 | 4.09E-02 | NM | NM |

**Supplemental Table 10: Potential GTF2I-regulated genes**

# Fold-change of RNAseq data is relative to CDKL5 isogenic controls, negative values indicate higher in CDKL5 p.(Arg59*). * Fold-change of qPCR data is relative to controls, negative values are lower in CDKL5 p.(Arg59*). WS; Williams-Beuren Syndrome, FC; Fold-change, CPM; Counts per million, LR; Log ratio, FDR; false discovery rate, NM; not measured.

| **Model system** | **Citation** | **Human Gene** | **Effect in model system** | **Human CDKL5 p.Arg59* neurons** | **RNAseq data** | | | **qPCR data** | |
| --- | --- | --- | --- | --- | --- | --- | --- | --- | --- |
|  |  |  |  |  | **logFC#** | **logCPM** | **FDR** | **FC*** | **P-value** |
| *Chd4*^loxP/loxP^ knockout mouse cortex (NuRD subunit) | Yamada *et al.* 2014 | *SYTL1* | ↓ | ↓ | -4.41 | 0.88 | 8.58E-04 | NM | NM |
|  |  | *NHLRC4* | ↓ | ↓ | -3.30 | 0.81 | 4.87E-02 | 1.10 | 2.99E-02 |
|  |  | *UBXN10* | ↓ | ↓ | -3.05 | 3.44 | 1.31E-02 | 0.50 | 2.88E-03 |
|  |  | *COL9A1* | ↓ | ↓ | -2.84 | 3.09 | 2.29E-04 | 1.12 | 5.70E-02 |
|  |  | *GEM* | ↓ | ↓ | -2.51 | 2.71 | 1.44E-02 | NM | NM |
|  |  | *WNT9A* | ↓ | ↓ | -2.22 | 3.70 | 1.46E-02 | NM | NM |
|  |  | *RGS11* | ↓ | ↓ | -2.06 | 0.97 | 6.13E-02 | NM | NM |
|  |  | *TEKT2* | ↓ | ↓ | -1.99 | 3.62 | 5.24E-02 | 0.90 | 1.13E-02 |
|  |  | *P4HA2* | ↓ | ↓ | -1.81 | 4.31 | 6.39E-04 | NM | NM |
|  |  | *LAMA5* | ↓ | ↓ | -1.77 | 4.92 | 4.81E-02 | 0.83 | 6.60E-03 |
|  |  | *CLIC1* | ↓ | ↓ | -1.54 | 6.88 | 2.28E-03 | 0.42 | 4.25E-03 |

**Supplemental Table 11: Potential NuRD-regulated genes**

# Fold-change of RNAseq data is relative to CDKL5 isogenic controls, negative values indicate higher in CDKL5 p.(Arg59*). * Fold-change of qPCR data is relative to controls, negative values are lower in CDKL5 p.(Arg59*). FC; Fold-change, CPM; Counts per million, LR; Log ratio, FDR; false discovery rate, NM; not measured.

**References**

1. Hornbeck PV, Zhang B, Murray B, Kornhauser JM, Latham V, Skrzypek E. PhosphoSitePlus, 2014: mutations, PTMs and recalibrations. *Nucleic Acids Res* 2015; **43**(Database issue)**:** D512-520.

2. Johnson JL, Yaron TM, Huntsman EM, Kerelsky A, Song J, Regev A *et al.* An atlas of substrate specificities for the human serine/threonine kinome. *Nature* 2023; **613**(7945)**:** 759-766.
